# *Foxm1* haploinsufficiency drives clonal hematopoiesis and promotes a stress-related transition to hematologic malignancy in mice

**DOI:** 10.1172/JCI163911

**Published:** 2023-08-01

**Authors:** Chunjie Yu, Yue Sheng, Fang Yu, Hongyu Ni, Alan Qiu, Yong Huang, Zhijian Qian

**Affiliations:** 1 Department of Medicine, UF Health Cancer Center, University of Florida, Gainesville, Florida, USA.; 2 Department of Biochemistry and Molecular Biology, University of Florida, Gainesville, Florida, USA.; 3 Department of Hematology, Second Xiangya Hospital, Changsha, Hunan, China.; 4 Department of Pathology, Cedars Sinai Medical Center, Los Angeles, California, USA.; 5 Department of Medicine, University of Virginia, Charlottesville, Virginia, USA.

**Keywords:** Hematology, Bone marrow differentiation, Hematopoietic stem cells, Leukemias

## Abstract

Clonal hematopoiesis plays a critical role in the initiation and development of hematologic malignancies. In patients with del(5q) myelodysplastic syndrome (MDS), the transcription factor FOXM1 is frequently downregulated in CD34^+^ cells. In this study, we demonstrated that *Foxm1* haploinsufficiency disturbed normal hematopoiesis and conferred a competitive repopulation advantage for a short period. However, it impaired the long-term self-renewal capacity of hematopoietic stem cells, recapitulating the phenotypes of abnormal hematopoietic stem cells observed in patients with MDS. Moreover, heterozygous inactivation of *Foxm1* led to an increase in DNA damage in hematopoietic stem/progenitor cells (HSPCs). *Foxm1* haploinsufficiency induced hematopoietic dysplasia in a mouse model with LPS-induced chronic inflammation and accelerated AML-ETO9a–mediated leukemogenesis. We have also identified Parp1, an important enzyme that responds to various types of DNA damage, as a target of Foxm1. *Foxm1* haploinsufficiency decreased the ability of HSPCs to efficiently repair DNA damage by downregulating *Parp1* expression. Our findings suggest that the downregulation of the Foxm1-Parp1 molecular axis may promote clonal hematopoiesis and reduce genome stability, contributing to del(5q) MDS pathogenesis.

## Graphical abstract

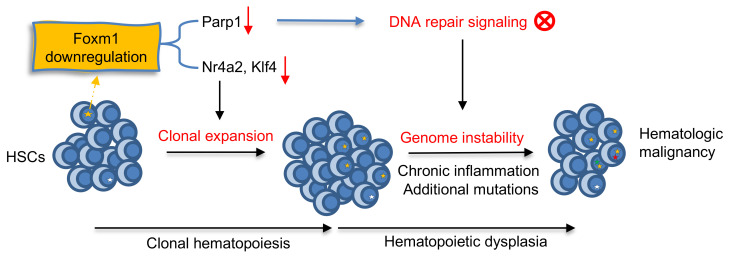

## Introduction

Normal hematopoietic stem cells (HSCs) can self-renew and produce all lineages of blood cells. The majority of HSCs are in a quiescent state under homeostasis, which is characterized by slow proliferation and rare differentiation (1–3). Myelodysplastic syndrome (MDS), a clonal HSC disease, is characterized by aberrant hematopoiesis with a defect in single or multiple lineages (4, 5). Clonal hematopoiesis (CH) is a highly prevalent process in elderly populations that greatly increases the risk of developing hematopoietic malignancies, including MDS, myeloproliferative neoplasms (MPNs), and acute myeloid leukemia (AML) (6, 7). It occurs when a mutated lineage of HSCs undergoes excessive expansion and dominates the process of differentiation (7). Recent studies have identified numerous factors that contribute to CH, including aging, environmental exposures, and germline genetic alteration (8–12). Aging, as a prevalent cause of CH, has been reported to cause accumulation of DNA damage and increase the risk of genetic instability (13–15).

Safeguarding genomic integrity is vital for functional HSCs to maintain hematopoietic homeostasis, considering that unrepaired genetic alteration in HSCs could be spread in the whole HSC pool and propagated to HPCs and mature cells. HSCs have a sophisticated DNA repair system, in which DNA lesions trigger DNA damage response (DDR) and activate different cellular responses, including DNA damage repair, cell cycle checkpoint, and so on, depending on the cell cycle phase and the physiological status of the HSCs with DNA lesions (16–18). The nuclear enzyme PARP-1, a member of the poly(ADP-ribose) polymerase (PARP) protein family, is activated by several forms of DNA damage to maintain genomic integrity (19, 20). PARP-1 has been reported to participate in DNA repair caused by irradiation, chemotherapy, and LPS-induced inflammation (21, 22). While PARP inhibitors (PARPi) have been approved for use in some solid tumors, including ovarian, breast, and prostate cancer (23–25), the effect of PARPi in hematopoietic malignancies is ambiguous (26). Studies have shown that long-term PARPi treatment for solid tumors, particularly ovarian cancer, may increase the risk of developing MDS and AML (27, 28).

Foxm1, a transcription factor in the Fox protein family, is involved in a variety of biological processes, such as cell cycle, cell growth, and cellular senescence, etc. (29). FOXM1 expression has been found to be elevated in various malignancies, such as liver, breast, prostate, and pancreas cancers, as well as others (30, 31). We previously showed that homozygous deletion of *Foxm1* disrupts normal hematopoiesis (32). The *FOXM1* expression level was significantly decreased in CD34^+^ cells from patients with del(5q) MDS (32). To determine whether *Foxm1* downregulation plays a pathogenic role in del(5q) MDS, we characterized the *Foxm1*-haploinsufficient mouse model with or without chronic stress. Notably, we found that *Foxm1* haploinsufficiency disrupted HSC homeostasis in young mice by exiting the quiescent stage and entering the cell cycle. Heterozygous deletion of *Foxm1* resulted in functional exhaustion of HSCs after a series of transplantation. Chronic inflammation stress induced malignant transformation of HSCs in *Foxm1* heterozygous mice, and Foxm1 haploinsufficiency promoted AML-ETO9A–induced (AE9a-induced) MPN/AML. Foxm1 haploinsufficiency significantly inhibited NR4a2 expression and disturbed the lysosome signaling pathway, which may partially contribute to Foxm1 haploinsufficiency–induced long-term HSC exhaustion. More importantly, we showed that Foxm1 acts as a gatekeeper of genome stability in hematopoietic stem/progenitor cells (HSPCs), at least partially through regulation of Parp1. In conclusion, our results suggest that Foxm1 downregulation may contribute to the development of CH and the malignant transformation of HSCs in hematopoietic diseases.

## Results

### Foxm1 haploinsufficiency disrupts HSC homeostasis by pushing HSCs to exit from quiescence and impairs long-term HSC repopulation ability.

**Figure 1:**
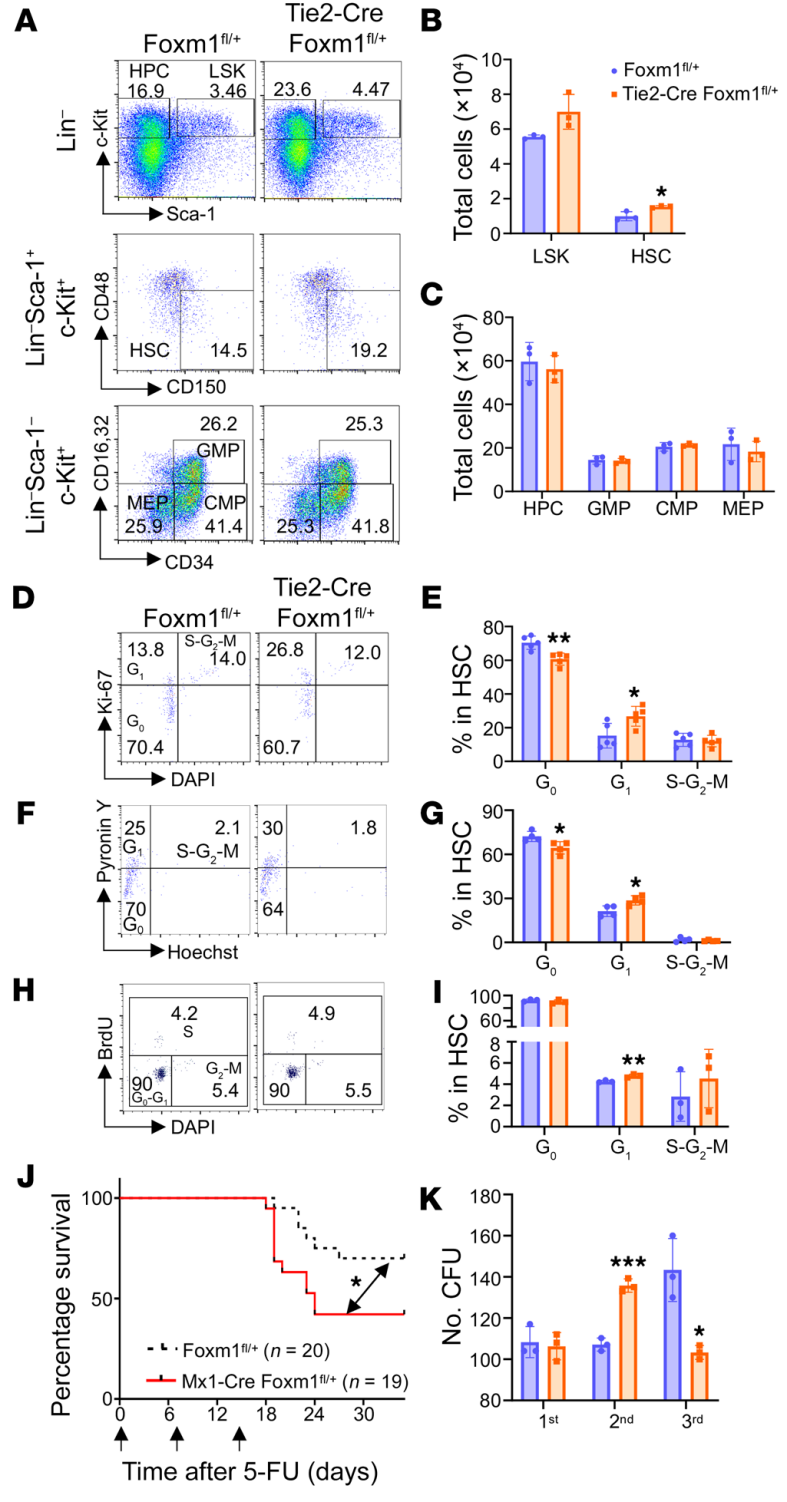
*Foxm1* haploinsufficiency promotes the expansion of HSCs by pushing HSC exit from quiescence. (**A**) Flow cytometric analysis of HPC, LSK, HSC, granulocyte-monocyte progenitor (GMP), common myeloid progenitor (CMP), and megakaryocyte-erythrocyte progenitor (MEP) populations in BM cells from *Foxm1*^fl/+^ mice or Tie2-Cre *Foxm1*^fl/+^ mice. (**B**) A total number of LSK cells and HSCs in BM from 8-week-old *Foxm1*^fl/+^ mice (*n* =3) and Tie2-Cre *Foxm1*^fl/+^ mice (*n* = 3). (**C**) Total number of HPC, GMP, CMP, and MEP cells in BM from 8-week-old *Foxm1*^fl/+^ mice (*n* = 3) or Tie2-Cre *Foxm1*^fl/+^ mice (*n* = 3). (**D** and **E**) Cell cycle analysis using DAPI (DNA content) and Ki-67 (proliferative cells) staining in HSCs, as determined by FACS. *n* = 5 mice for each group. (**F** and **G**) Cell cycle analysis using Hoechst (DNA dye) and Pyronin Y (RNA dye) staining in HSCs, as determined by FACS. *n* =4 mice for each group. (**H** and **I**) Cell cycle analysis using DAPI (DNA content) and BrdU (cells in S phase) staining in HSCs, as determined by FACS. *n* =3 mice for each group. (**J**) Kaplan-Meier survival curve of *Foxm1*^fl/+^ (*n* = 20) and Mx1-Cre *Foxm1*^fl/+^ mice (*n* = 19) after multiple injections (upward arrows) of 5-FU (150 mg/kg body weight). The double-headed arrow denotes the difference (*P* value) between two groups as indicated. Log-rank (Mantel-Cox) test. (**K**) The colony-forming assay shows repopulation ability. 5 × 10^4^ cell input for the first round, and 1.5 × 10^5^ cell input for the second round and third rounds. Data are representative of at least 2 independent experiments and expressed as mean ± SD. *P ≤ 0.05, **P < 0.01, ***P < 0.001; 2-tailed Student’s *t* test or log-rank (Mantel-Cox) test for survival curve.

To investigate whether Foxm1 downregulation affects hematopoiesis, we employed the *Foxm1*-haploinsufficient mouse model. The *Foxm1*^fl/fl^ mice with loxP-flanked *Foxm1* alleles were crossed with Tie2-Cre transgenic mice to generate Tie2-Cre *Foxm1*^fl/+^ mice, in which 1 allele of the *Foxm1* gene was deleted in HSPCs. Both *Foxm1*^fl/+^ and Tie2-Cre *Foxm1*^fl/+^ mice at 8 weeks of age displayed normal hematological parameters (Supplemental Figure 1, A–D; supplemental material available online with this article; https://doi.org/10.1172/JCI163911DS1). The frequencies of mature myeloid cells, red cells, and B cells in BM and spleen were comparable, while that of immature B cells (B220^+^IgM^–^) was significantly decreased in *Foxm1*-haploinsufficient mice (Supplemental Figure 1, H–L). In addition, the frequencies of hematopoietic progenitor cells (HPCs) as well as subpopulations of myeloid progenitor cells, including common myeloid progenitors(CMPs), granulocyte-monocyte progenitors(GMPs), and megakaryocyte-erythroid progenitors(MEPs), were comparable in *Foxm1*^fl/+^ or Tie2-Cre *Foxm1*^fl/+^ mice, while the frequencies of stem cell-enriched population (LSKs) (Lin^–^c-Kit^+^Sca-1^+^) and HSCs (Lin^–^c-Kit^+^Sca-1^+^CD48^–^CD150^+^) were slightly increased, as determined by flow cytometric analysis (Figure 1A and Supplemental Figure 1F). Interestingly, we observed that the total number of HSCs but not HPCs (Lin^–^c-Kit^+^Sca-1^–^) was increased significantly in Tie2-Cre *Foxm1*^fl/+^ mice as compared with *Foxm1*^fl/+^ (Figure 1, B and C).

Dormant HSCs reside in the G_0_ phase of the cell cycle and enter the cell cycle for proliferation. We examined whether the observed expansion of HSCs was associated with changes in cell cycle status. Flow cytometric analysis of cell cycle by Ki67/DAPI and Pyronin-Y/Hoechst staining revealed a significant decrease in the frequency of cells in the G_0_ phase and a reciprocal increase in G_1_ phase cells in *Foxm1* heterozygous HSCs compared with cells from the control group (Figure 1, D–G). BrdU incorporation assay showed that the frequency of S phase cells was slightly increased in *Foxm1* heterozygous HSCs (Figure 1, H and I). In contrast to the cell cycle alterations, *Foxm1* haploinsufficiency has a marginal effect on the viability of HSCs (Supplemental Figure 1G). To exclude the influence of Tie2-Cre, we characterized mice with Mx1-Cre–mediated *Foxm1* KO. We observed similar phenotypes in Mx1-Cre mice (Supplemental Figure 2). These results suggest that Foxm1 plays a role in HSC homeostasis by maintaining quiescence but does not affect cell survival.

As *Foxm1* haploinsufficiency leads to a significant increase in cycling HSCs, we next examined the reconstitution capacity of HSCs. 5-FU was administered i.p. weekly to *Foxm1* heterozygous mice and littermate control mice to target proliferating cells in BM and activate quiescent HSCs to replenish the hematopoietic system. Sequential treatment with 5-FU can lead to HSC exhaustion and, ultimately, BM failure and death of the animals. Surprisingly, *Foxm1* heterozygous mice had a significantly shorter life span compared with that of control mice (Figure 1J). Consistent with this result, we observed a defective repopulation capacity in *Foxm1* heterozygous KO BM cells in vitro determined by serial replating assays. After the first plating, we observed comparable numbers of colonies. However, in the second plating, *Foxm1* heterozygous HSPCs gave rise to significantly more colonies, while they produced significantly fewer colonies in the third plating compared with control HSPCs (Figure 1K). These results suggest that *Foxm1* haploinsufficiency initially enhances the short-term repopulation ability of HSPCs but eventually leads to exhaustion of their repopulation capacity in vitro.

### Foxm1 haploinsufficiency impaired repopulating potential of HSCs.

**Figure 2:**
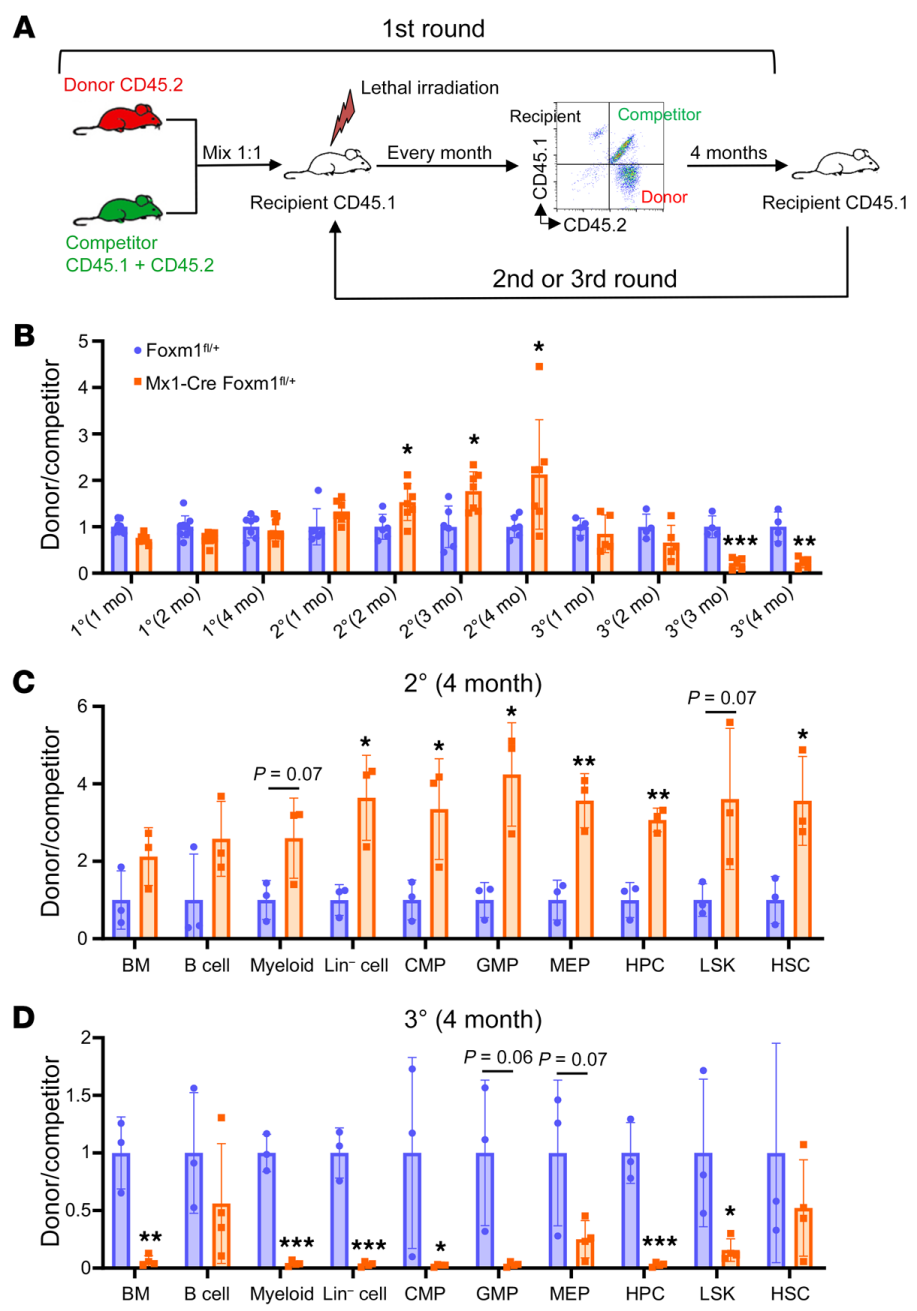
*Foxm1* haploinsufficiency results in enhanced reconstitution capacity but eventually leads to HSC pool exhaustion due to attrition. (**A**) Schematic depiction of the competitive transplantation assay. (**B**) The relative ratio of donor-derived cells (CD45.1^–^CD45.2^+^) to competitor-derived cells (CD45.1^+^CD45.2^+^) in peripheral blood. For WT group, *n* = 7–9, 6–10, and 4 for the first (1°), second (2°), and third (3°) transplantation, respectively; for *Foxm1* HET group, *n* = 9, 7–9, and 5 for 1°, 2°, and 3° transplantation, respectively. (**C**) The ratio of donor-derived cells to competitor-derived cells in different populations, as indicated in BM at the fourth month after the second transplantation. *n* = 3 for both WT and *Foxm1* HET groups. (**D**) The ratio of donor-derived cells to competitor-derived cells in different populations, as indicated in BM at the fourth month after the third transplantation. *n* = 3 for WT group and *n* = 4 for *Foxm1* HET group. Data are representative of 2 independent experiments and expressed as mean ± SD. *P < 0.05, **P < 0.01, ***P < 0.001; 2-tailed Student’s *t* test.

To further investigate whether *Foxm1* haploinsufficiency influences the long-term repopulating potential of HSPCs in vivo, we performed a competitive repopulation assay. Whole BM cells from *Foxm1*^fl/+^ or Tie2-Cre *Foxm1*^fl/+^ mice (CD45.2^+^) were transplanted along with an equal number of CD45.1^+^CD45.2^+^ WT BM cells into lethally irradiated CD45.1^+^ WT recipients (Figure 2A). For serial BM transplantation, equal numbers of BM cells from each group 4 months after transplantation were transplanted into secondary or tertiary recipients. Donor-derived peripheral blood (PB) cells and WT competitor-derived PB cells were determined by flow cytometry each month. Strikingly, we observed an increased contribution of *Foxm1*-haploinsufficient BM cells to PB during the first 2 transplantation cycles (Figure 2B). In addition, we evaluated HSPCs at the fourth month after the second transplantation. Flow cytometry analysis revealed that *Foxm1*-haploinsufficient HSPCs outcompeted their WT counterparts, suggesting that *Foxm1*-haploinsufficient HSPCs cells have a competitive self-renewal advantage over the control HSPCs (Figure 2C). However, in the third transplantation, we observed a significant decrease in the proportion of PB cells derived from *Foxm1*-haploinsufficient HSPCs (Figure 2B). In the BM of recipients, the ratio of *Foxm1* heterozygous cells was also significantly decreased in HSPCs and downstream myeloid progenitor cells, along with mature myeloid cells (Figure 2D). To further validate the “up-and-down” effect conferred by *Foxm1* haploinsufficiency in the setting of competitive repopulation, we used Mxl-Cre *Foxm1*^fl/+^ mice to examine long-term repopulation potential. Consistently, *Foxm1*-haploinsufficient HSPCs exhibited a robust repopulation ability in the first round of transplantation, while they showed a remarkable decrease of repopulation ability in PB and BM in the secondary transplantation (Supplemental Figure 3).

### Foxm1-haploinsufficient mice developed hematopoietic dysplasia under long-term chronic stress.

**Figure 3:**
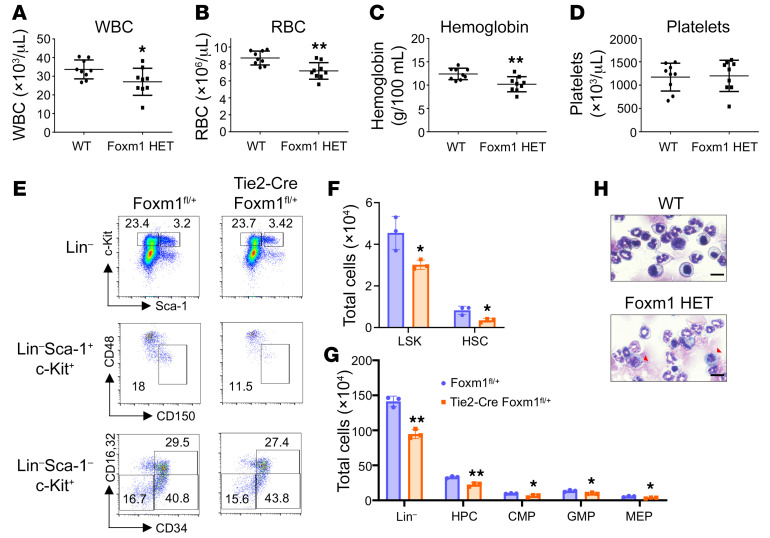
Foxm1 is needed to maintain HSC homeostasis in response to chronic inflammation. (**A**–**D**) Lethally irradiated mice reconstituted with BM cells from *Foxm1*^fl/+^ mice (*n* = 9) or Tie2-Cre *Foxm1*^fl/+^ mice (*n* = 9). LPS was injected twice per week for 2 months. Complete blood count of peripheral blood was assessed every month after LPS challenge. (**A**) White blood cells, (**B**) red blood cells, (**C**) hemoglobin, and (**D**) platelets were analyzed. (**E**) Flow cytometric analysis shows the frequency of HPC, LSK, HSC, GMP, CMP, and MEP populations from chimeric mice injected with 1 mg/kg LPS as indicated. (**F** and **G**) Total cells were assessed for BM cells and Lin^–^, LSK, HSC, HPC, GMP, CMP, and MEP populations. *n* = 3 for each group. Data are presented as mean ± SD. *P < 0.05, **P < 0.01; 2-tailed Student’s *t* test. (**H**) Giemsa-Wright staining of BM cells from WT or *Foxm1* HET BMT mice treated with LPS for 2 months. Red arrowheads indicate nucleated red blood cells with nuclear irregularities. Scale bar: 50 μm.

Inflammation has been reported as one of the factors that contributes to hematopoietic disorders (33, 34). Prolonged exposure to inflammatory stimuli has been shown to have a long-lived impairment on HSC function and accelerate hematopoiesis aging (35). Individuals usually undergo chronic low-level inflammation during aging, which may also promote age-associated hematologic diseases (34). To investigate the role of Foxm1 in hematopoiesis under inflammatory conditions, we administered long-term low-dose injections of LPS to mimic a chronic inflammation environment in animals (36, 37). The recipient mice transplanted with BM cells from *Foxm1* heterozygous mice or littermate control mice were treated with a low dose of LPS twice every week. After 2 months of injections, we found that the mice transplanted with BM cells from *Foxm1* heterozygous mice developed multilineage cytopenia, characterized by significantly decreased white blood cells, red blood cells, and hemoglobin, while the number of platelets did not change (Figure 3, A–D). In addition, we noted the presence of nucleated red blood cells with nuclear irregularities (Figure 3H), which is a frequently observed morphological feature in patients with MDS. We subsequently performed flow cytometric analysis on the mice. The results indicate that the total numbers of HSCs and progenitors were significantly decreased in *Foxm1*-haploinsufficient mice compared with that in control WT mice (Figure 3, E–G). Taken together, these data suggest that *Foxm1* haploinsufficiency is more prone to develop hematopoietic malignancy under inflammatory stress.

### The molecular pathways that are deregulated in Foxm1 heterozygous HSPCs.

**Figure 4:**
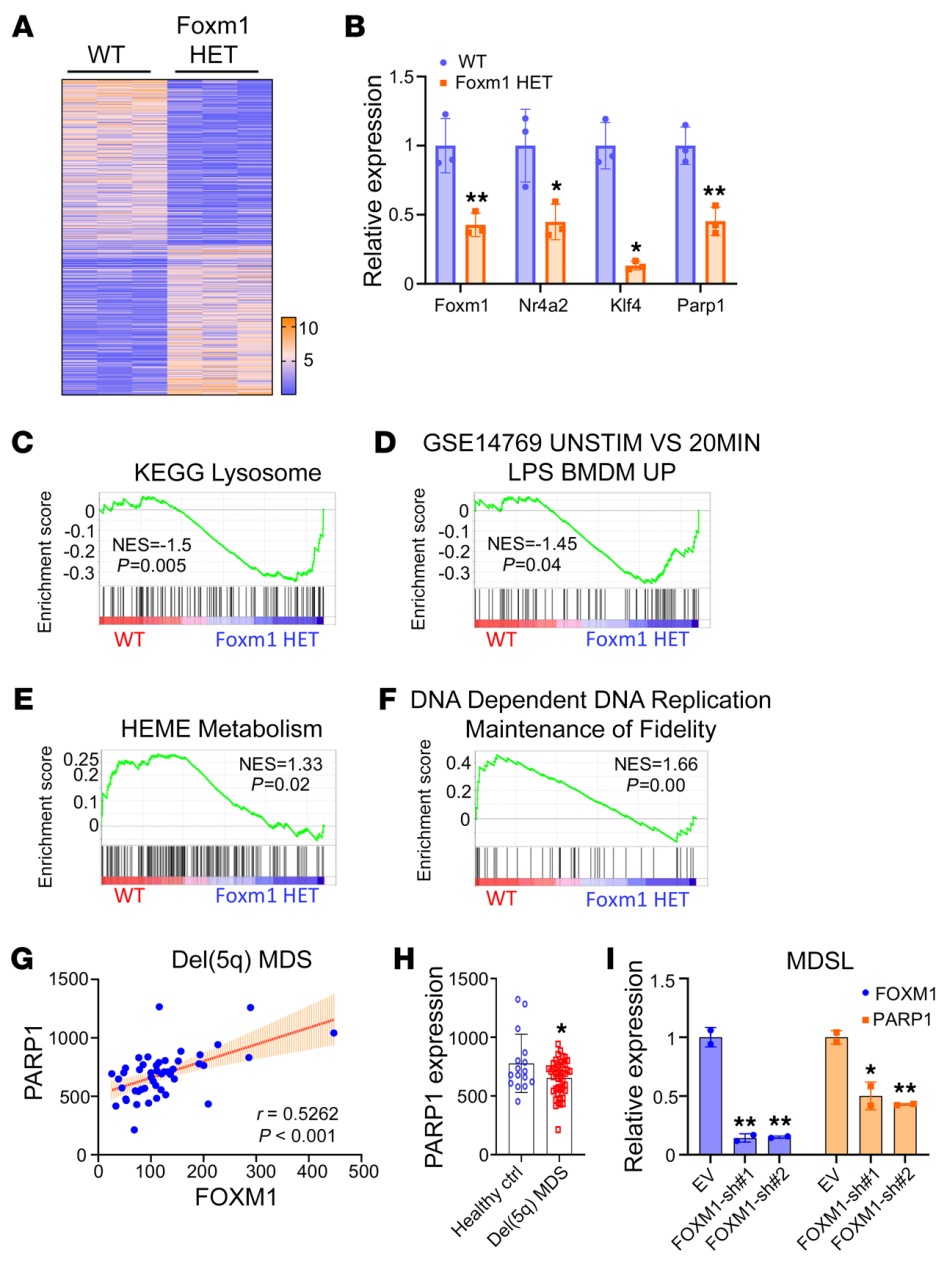
Foxm1 regulates multiple signaling pathways in HSCs. (**A**) Heatmap of expression profiles for genes with *P* < 0.05 (576 genes). (**B**) RT-PCR analysis shows the expression level of genes involved in stem cell functions and DNA damage repair pathway in HSCs from WT(*n* = 3) or *Foxm1* HET(*n* = 3) mice. (**C** and **D**) GSEA plots show, respectively, a positive association with “KEGG Lysosome” and “GSE14769 UNSTIM VS 20MIN LPS BMDM UP” in *Foxm1* HET HSCs compared with WT HSCs. (**E** and **F**) GSEA plots show a negative association with “HEME Metabolism” and “DNA Dependent DNA Replication Maintenance of Fidelity” in *Foxm1* HET HSCs compared with WT HSCs. (**G**) Correlation between *FOXM1* and *PARP1* in MDS with deletion of chromosome 5q [del(5q) MDS] (*n* = 47). *P* value was calculated by Spearman’s *r* correlation. (**H**) Microarray analysis of *PARP1* in CD34^+^ cells from individuals in the healthy control group (*n* =16) or from patients with del(5q) MDS (*n* = 43). (**I**) RT-PCR analysis of *FOXM1* and *PARP1* in MDSL cells transduced *FOXM1* shRNAs and control vector; the results were normalized to *ACTB* expression. Data are presented as mean ± SD. *P < 0.05, **P < 0.01, 2-tailed Student’s *t* test.

To determine the mechanism by which *Foxm1* haploinsufficiency impairs HSC functions, we performed RNA-Seq analysis on long-term HSCs isolated from *Foxm1* heterozygous mice and WT mice at 8 weeks of age. The heatmap showed that 273 genes were upregulated and 303 genes were downregulated in *Foxm1* heterozygous LT-HSCs (Figure 4A). Nr4a2, a key regulator of HSC quiescence (38), was significantly downregulated as a result of the heterozygous deletion of *Foxm1* in HSCs (Figure 4B). Gene enrichment analysis (GSEA) showed that the lysosome signaling pathway, which has been reported to regulate HSC quiescence and potency (39), was significantly upregulated in *Foxm1* heterozygous HSCs (Figure 4C). These data suggest that the downregulation of *Nr4a2* and the overactivity of the lysosome signaling pathway may contribute to HSC functional defects induced by *Foxm1* haploinsufficiency. Additionally, the *Klf4* gene, which is involved in the regulation of hematopoiesis (40–42), was also markedly downregulated in *Foxm1* heterozygous HSCs (Figure 4B). In line with the results from the LPS treatment assay (Figure 3), GSEA showed that the genes upregulated in macrophage cells with LPS stimulation were significantly enriched in *Foxm1* heterozygous HSCs (Figure 4D). The genes associated with HEME (a cofactor consisting of iron and porphyrin) metabolism and erythroblast differentiation were significantly downregulated in *Foxm1* heterozygous HSCs (Figure 4E and Supplemental Figure 4A), which may partially explain the reduction of erythroid in chronic inflammation conditions (Figure 3, B and C). Moreover, we found that the genes involved in the maintenance of fidelity in the process of DNA replication were downregulated in *Foxm1* heterozygous HSCs (Figure 4F). FOXM1 was previously reported to be involved in the DNA repair pathway in breast cancer cells, pancreatic cancer cells, and glioma cells (43–45). However, no alterations in the expression of other known downstream targets of Foxm1 involved in DNA repair pathways were detected in *Foxm1* heterozygous HSCs (Supplemental Figure 4B). Of interest, the expression of *Parp1*, an important DNA damage sensor (46, 47), was significantly decreased in *Foxm1* heterozygous HSCs (Figure 4B). Analysis of the public database GSE19429 (48) revealed that *PARP1* has a slightly positive correlation with *FOXM1* in CD34^+^ cells from patients with del(5q) MDS (*P* < 0.001) (Figure 4G). Indeed, *PARP1* displayed a relatively low expression level in CD34^+^ cells from patients with del(5q) MDS compared with healthy individuals (Figure 4H). In line with public data, we showed that *FOXM1* knockdown lead to significant downregulation of *PARP1* in the MDSL cell line (Figure 4I), which was derived from a patient with MDS (49). Moreover, we also observed a lower level of *Parp1* in 5-FU–enriched progenitor and stem cell populations from *Foxm1* heterozygous mice injected with 5-FU for 5 days (Supplemental Figure 4C). These findings suggest that Foxm1 governs a distinctive cluster of DNA repair genes in HSPCs and that the function of *Foxm1* haploinsufficiency in HSPCs is mediated by multiple downstream pathways.

### Foxm1-haploinsufficient HSPCs show an impaired DNA damage repair.

**Figure 5:**
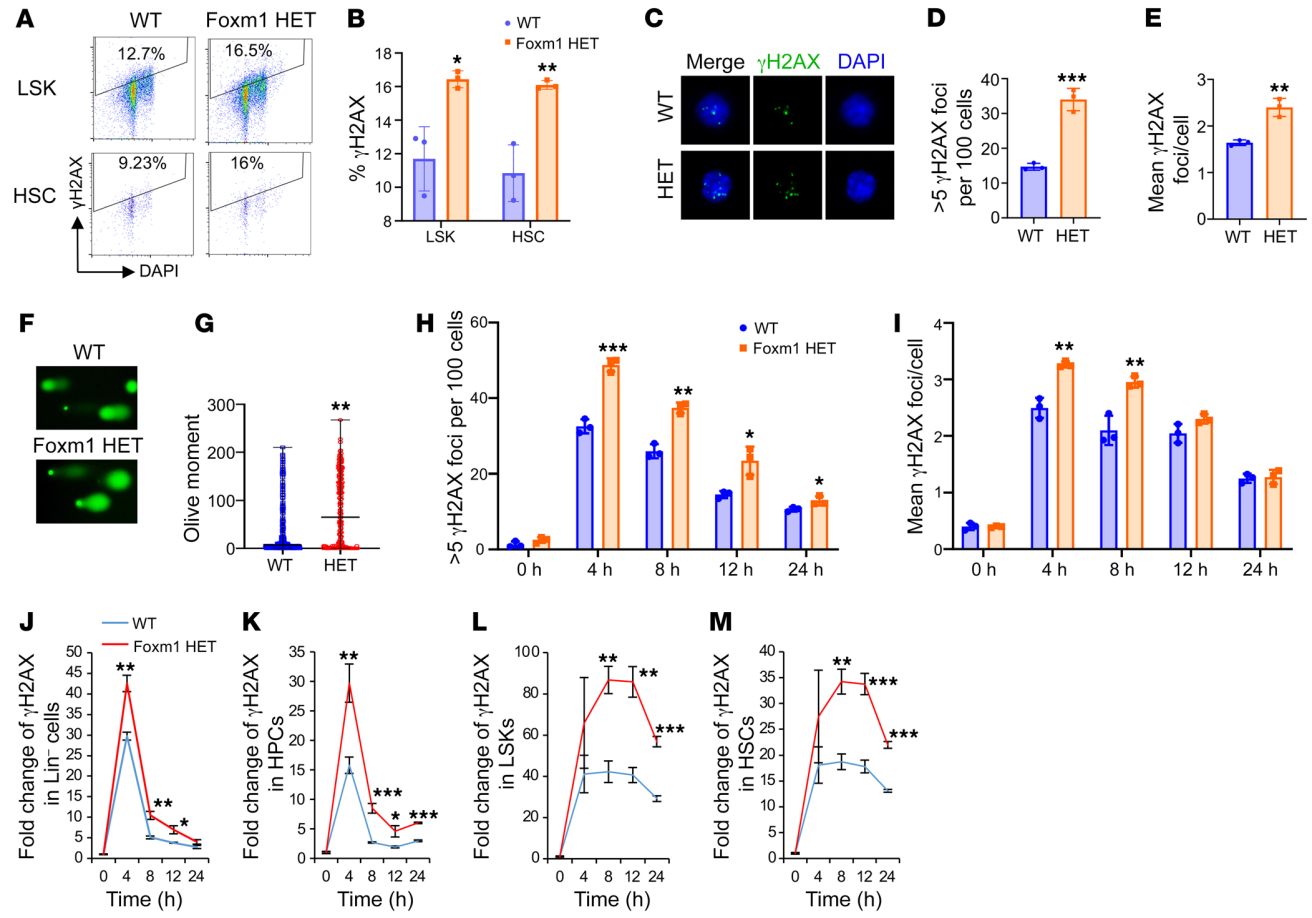
*Foxm1* haploinsufficiency results in a defect in DNA damage repair in HSPCs. (**A**) Representative flow cytometric plots show the percentage of γH2AX^+^ cells in LSK and HSC populations from Tie2-Cre *Foxm1*^fl/+^ mice (*n* = 3) and control mice (*n* = 3) 24 hours after LPS injection. (**B**) Histogram shows the percentage of γH2AX^+^ cells in LSK and HSC populations. (**C**) Representative images of γH2AX foci in Lin^–^c-Kit^+^ BM cells isolated from Tie2-Cre *Foxm1*^fl/+^ and control mice after LPS injection. Magnification: ×63 oil for **C** and ×20 for **F**. (**D**) Quantification of the number of cells with more 5 γH2AX foci per 100 Lin^–^c-Kit^+^ BM cells from the mice injected with LPS after 24 hours. *n* = 3 for each group. (**E**) Quantification of the mean γH2AX foci/cells in Lin^–^c-Kit^+^ BM cells from the mice injected with LPS after 24 hours. *n* = 3 for each group. (**F**) Representative images of the alkaline comet assay detecting DNA damage in Lin^–^c-Kit^+^ BM cells from mice injected with LPS after 24 hours. (**G**) Quantitative results of the tail olive moment for the alkaline comet assay. Three independent experiments were performed, and the results presented are the pooled data from all 3 experiments. (**H**) Quantification of the number of cells with more 5 γH2AX foci per 100 Lin^–^ BM cells exposed to irradiation (0.5 Gy) at different time points. *n* = 3 for each group. (**I**) Quantification of the mean γH2AX foci/cell in Lin^–^ BM cells exposed to irradiation (0.5 Gy) at different time points. *n* = 3 for each group. (**J**–**M**) Kinetics of γH2AX^+^ cells in different populations from the cells exposed to 0.5 Gy irradiation over the indicated period. *n* = 3 for each group. Data are representative of at least 2 independent experiments and expressed as mean ± SD. *P < 0.05, **P < 0.01, ***P < 0.001; 2-tailed Student’s *t* test.

As *Foxm1* deficiency leads to the downregulation of several critical genes involved in DNA repair, we next determined whether Foxm1 loss affects DDR in HSPCs. We treated *Foxm1* heterozygous mice and control littermates with LPS. Intracellular flow cytometric analysis revealed that *Foxm1*-haploinsufficient cells exhibited increased levels of γH2AX, a marker of unrepaired double-strand DNA breaks, in both LSKs and HSCs from *Foxm1* heterozygous mice compared with those in control mice (Figure 5, A and B). Consistently, increased γH2AX foci were observed in Lin^–^c-Kit^+^ cells from *Foxm1* heterozygous mice compared with control mice (Figure 5, C–E). Meanwhile, we also performed alkaline comet assay, a commonly used method to measure the level of cellular DNA double-stand DNA breaks. Lin^–^c-Kit^+^ cells from *Foxm1* heterozygous mice showed a significant increase in the tail olive moment, a measure of DNA double-stand DNA breaks, compared with control mice after LPS treatment. (Figure 5, F and G). To impose a DNA replication challenge on HSCs, we treated the mice with 5-FU, which kills cycling hematopoietic cells and thereby drives quiescent HSCs to enter the cell cycle and proliferate. Consistent with the results in LPS-treated mice, a significantly elevated level of γH2AX in populations of Lin^–^ cells, HPCs (Lin^–^Sca-1^–^c-Kit^+^), and LSKs (Lin^–^Sca-1^+^c-Kit^+^) was detected by flow cytometric analysis in *Foxm1* heterozygous mice compared with control mice (Supplemental Figure 5, A–D). Immunostaining of γH2AX revealed increased γH2AX foci in Lin^–^ cells from *Foxm1* heterozygous mice compared with controls (Supplemental Figure 5, E and F).

To further investigate the effect of Foxm1 haploinsufficiency on DNA damage repair during hematopoiesis, we next examined the dynamics of γH2AX foci in Lin^–^ cells after mice were exposed to 0.5 Gy irradiation. The number of γH2AX foci dramatically increased in both *Foxm1*-haploinsufficient and control Lin^–^ cells 4 hours after irradiation and then gradually decreased over time (Figure 5, H and I, and Supplemental Figure 5G). In comparison, *Foxm1*-haploinsufficient cells had a higher level of γH2AX foci compared with control cells during repair of irradiation-induced DNA damage. Consistent with immunostaining results, intracellular flow cytometric analysis showed a peak in γH2AX level at 4 hours, which gradually declined in subpopulations of HSPCs. (Figure 5, J–M). However, *Foxm1* haploinsufficiency led to significantly higher levels of γH2AX in Lin^–^, HPCs, LSKs, and HSCs compared with control cells (Figure 5, J–M), indicating a delay in DNA damage repair. Taken together, these results suggest that *Foxm1* haploinsufficiency decreases the capacity of HSPCs to efficiently repair irradiation-induced DNA damage.

### Parp1 rescues the defect of DDR induced by Foxm1 haploinsufficiency.

**Figure 6:**
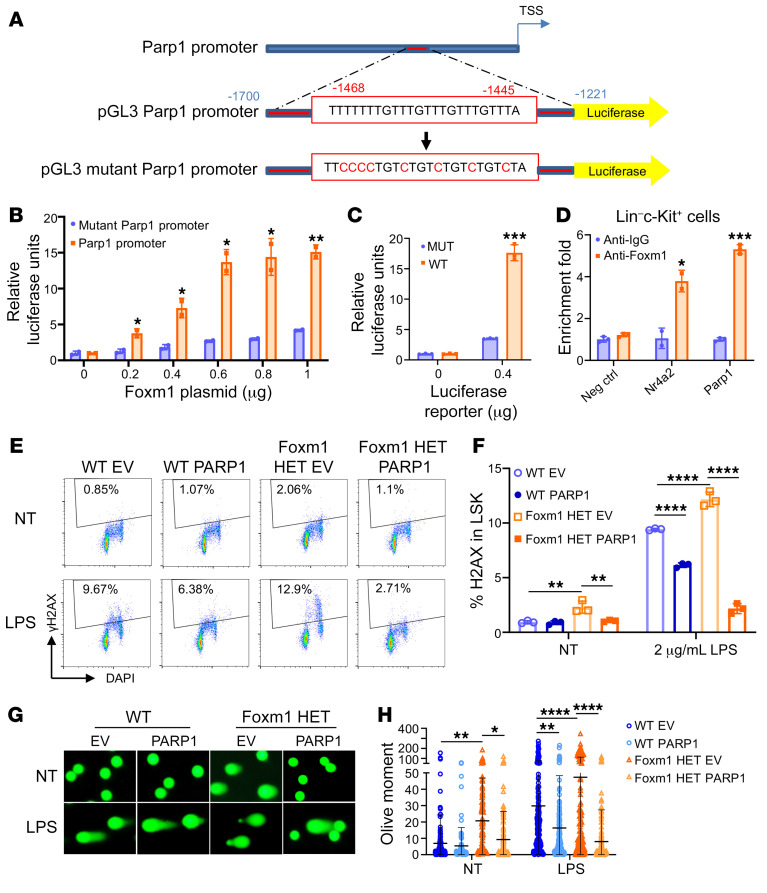
Overexpression of PARP1 rescues the defect of DNA damage repair caused by *Foxm1* haploinsufficiency in HSPCs upon LPS challenge. (**A**) Predicted binding site of Foxm1 in the promoter of Parp1 and the mutant binding site were subcloned to luciferase reporter vector pGL3. (**B**) Luciferase assay in 293T cells. All relative luciferase activity values are corrected for cotransfected Renilla activity. Data are from 3 independent experiments. (**C**) Luciferase assay in K562 cells with mouse Foxm1 expression. (**D**) Cut&Run assay was performed using purified Lin^–^c-Kit^+^ BM cells. RT-PCR shows the enrichment of Foxm1 at the promoter of *Parp1* in Lin^–^c-Kit^+^ HPCs. The intergenic region of *Actb* was used as a negative control. Data are from 2 independent experiments. (**E**) Representative flow cytometric plots show the percentage of γH2AX^+^ cells in LSK population from Lin^–^c-Kit^+^ BM cells transduced Parp1 or MSCV empty vector with or without LPS treatment. (**F**) Histogram shows the percentage of γH2AX^+^ cells in LSK population. (**G**) Representative images of the alkaline comet assay using purified Lin^–^c-Kit^+^ BM cells treated with LPS for 24 hours. Original magnifcation ×20. (**H**) Quantification of the tail olive moment for comet assays. Data are representative of at least 2 independent experiments and presented as mean ± SD. *P < 0.05, **P < 0.01, ***P < 0.001, ****P < 0.0001; 2-tailed Student’s *t* test (**B**–**D**) and 2-way ANOVA with Tukey’s multiple-comparison test (**F** and **H**).

We have shown that Parp1 is a potential downstream target of Foxm1 (Figure 4, B and I, and Supplemental Figure 4C). To determine whether transcriptional activation of *Parp1* is directly regulated by Foxm1, we searched for the Foxm1-binding sites in the *Parp1* promoter region using the PROMO database (based on version 8.3 of TRANSFAC; https://alggen.lsi.upc.es/cgi-bin/promo_v3/promo/promoinit.cgi?dirDB=TF_8.3) (50, 51). A putative binding site for Foxm1 was identified upstream of the *Parp1* transcription start site (–1,468 to –1,445 base pair) (Figure 6A). The dual-luciferase reporter assay was performed using 293T cells and K562 cells expressing either the WT *Parp1* promoter or a mutant promoter with mutations of the predicted Foxm1 binding site (Figure 6, B and C). The result showed that the luciferase activity of a construct containing the putative binding site but not the mutated binding site was activated by Foxm1 overexpression (Figure 6, B and C), suggesting that the putative Foxm1 binding site in the *Parp1* promoter is required for Foxm1-mediated activation of Parp1. We next performed a Cut&Run assay with mouse primary Lin^–^c-Kit^+^ BM cells. We previously showed that Foxm1 directly binds to the promoter of *Nr4a2* (32). Our result showed that the Foxm1 binding affinity was significantly enriched at the *Nr4a2* promoter and *Parp1* promoter regions containing the putative Foxm1 binding sites (Figure 6D), indicating that Foxm1 binds directly to the *Parp1* promoter and regulates its transcription.

We next determined whether Parp1 is a critical mediator of Foxm1 in regulating DDR. Lin^–^c-Kit^+^ BM cells from Foxm1 heterozygous mice or WT mice were subjected to overexpression of the human *PARP1* gene and treated with LPS for 24 hours. Flow cytometric analysis of γH2AX level in LSK and Lin^–^ cells indicated that ectopic expression of *PARP1* significantly rescued the defect of DNA damage repair as a result of *Foxm1* haploinsufficiency (Figure 6, E and F, and Supplemental Figure 6, E and F). Meanwhile, comet assays were performed on isolated Lin^–^c-Kit^+^ cells following 24 hours of LPS treatment. The results showed that the tail olive moment was reduced in PARP1 overexpressed cells compared with empty vector cells, validating the rescue for the defect of DNA damage repair caused by *Foxm1* haploinsufficiency (Figure 6, G and H). These findings suggest that Parp1 may play an important role in Foxm1-mediated DNA damage repair in HSPCs.

### Foxm1 haploinsufficiency promotes AE9a-mediated leukemogenesis.

**Figure 7:**
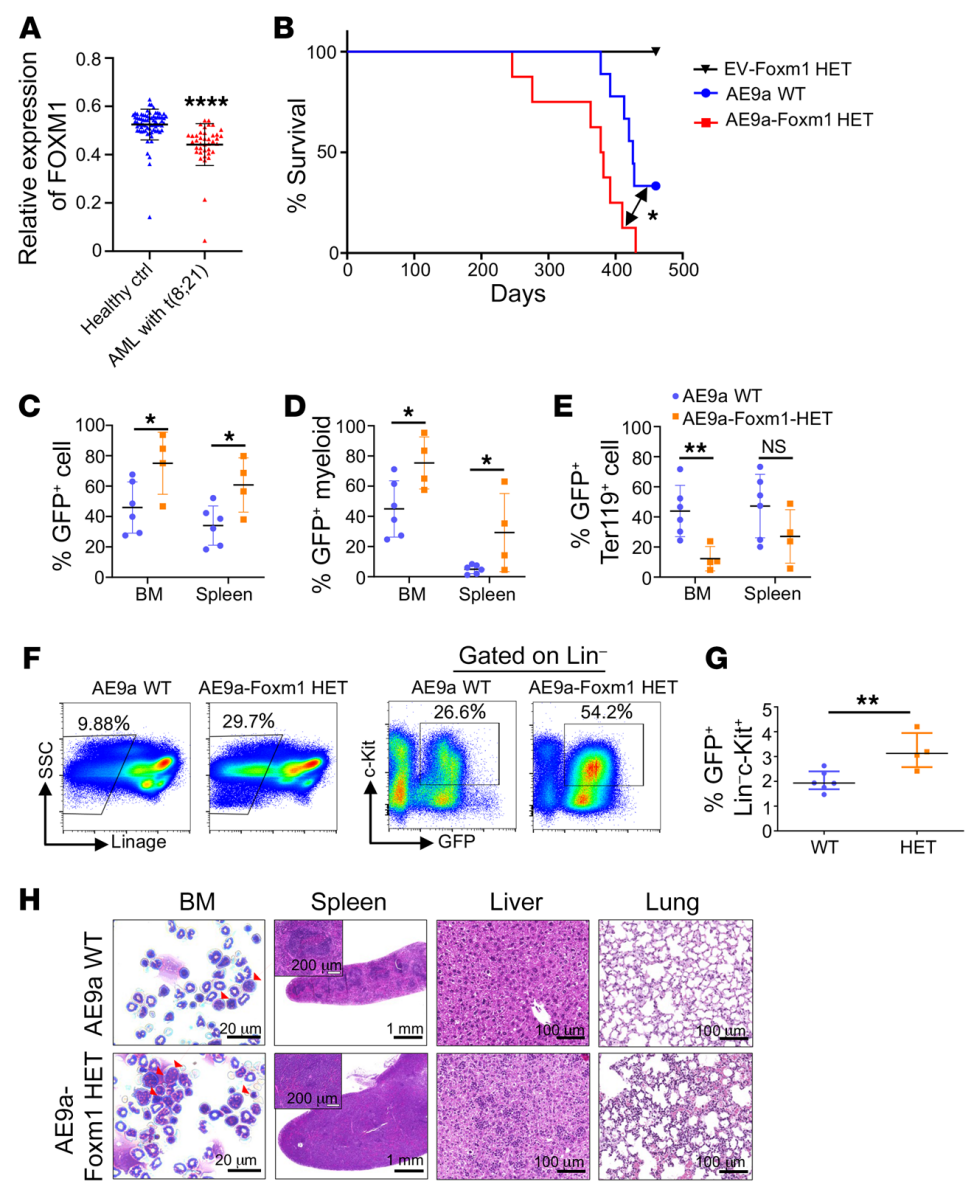
*Foxm1* haploinsufficiency promotes AML1-ETO9a–induced MPN/AML in mice. (**A**) Comparison of expression levels in patients with t(8;21) AML (ref. 66) (*n* = 40) and healthy individuals from the control group (*n* = 74) using data derived from GSE13159. (**B**) Kaplan-Meier survival analysis of EV-*Foxm1* HET mice (*n* = 7), WT AML1-ETO9a (AE9a) mice (*n* = 9), and AE9a-*Foxm1* HET mice (*n* = 8). Log-rank test. The double-headed arrow denotes the difference (*P* value) between two groups as indicated. (**C**) The frequency of GFP^+^ cells in BM and spleen. (**D** and **E**) The frequency of GFP^+^ myeloid cells (**D**) and GFP^+^ red blood cells (**E**) in BM cells and splenic cells. *n* = 6 for WT AE9a group, *n* = 4 for AE9a-Foxm1 HET group. (**F**) Flow cytometric analysis of the percentage of Lin^–^ cells and GFP^+^Lin^–^c-Kit^+^ cells in BM. (**G**) Quantification of the percentage of GFP^+^Lin^–^c-Kit^+^ cells. *n* = 6 for WT AE9a mice, *n* = 4 for AE9a-*Foxm1* HET mice. (**H**) Wright-Giemsa–stained BM cells and H&E-stained spleen, liver, and lung from WT AE9a and AE9a-Foxm1 HET mice. Data are presented as mean ± SD. *P <0.05, **P < 0.01, ****P < 0.0001; 2-tailed Student’s *t* test, or log-rank (Mantel-Cox) test for survival curve. Scale bar: 20 μm (BM); 1 mm (spleen); 200 μm (spleen inset); 100 μm (liver and lung).

Based on the analysis of the GSE13159 data set from Microarray Innovations in Leukemia (52, 53), we found that *Foxm1* was significantly downregulated in patients with t(8;21) AML (Figure 7A). To test the hypothesis that Foxm1 downregulation may promote AE9a-induced leukemogenesis, we overexpressed AE9a in BM cells from *Foxm1* heterozygous or control mice followed by transplantation in sublethally irradiated recipient mice. Indeed, *Foxm1* haploinsufficiency promoted the disease progression induced by AE9a (Figure 7B). Majority of AE9a-expressing mice developed MPN/AML-like disease. Compared with the WT chimeric mice, the AE9a-expressing cells (GFP^+^) expanded to a greater degree in BM and spleen in *Foxm1* heterozygous chimeric mice (Figure 7C). The frequency of myeloid cells was increased while red blood cells were decreased in *Foxm1* heterozygous AE9a chimeric mice as compared with WT AE9a chimeric mice (Figure 7, D and E, and Supplemental Figure 7, D and E). Moreover, platelets were significantly decreased in *Foxm1* heterozygous AE9a chimeric mice (Supplemental Figure 7C). Flow cytometric analysis revealed a greater accumulation of AE9a blast cells (GFP^+^Lin^–^c-Kit^+^) in the BM of *Foxm1* heterozygous AE9a chimeric mice compared with WT AE9a chimeric mice (Figure 7, F and G). Morphological analysis revealed more blast cells in the BM as well as leukemia cell infiltration in the spleens, livers, and lungs of *Foxm1* heterozygous AE9a mice (Figure 7H). We also monitored the mice transplanted with *Foxm1* heterozygous BM cells with empty vector. There were no observable phenotypes associated with AE9a-induced MPN/AML, indicating that the phenotypes observed in AE9a-transduced mice were indeed caused by the expression of AE9a (Supplemental Figure 7, A–C). Taken together, these data suggest that heterozygous loss of *Foxm1* accelerates AE9a-induced leukemogenesis in mice.

## Discussion

CH is a common aging process in which the mutant HSPCs acquire a growth advantage over normal HSPCs, resulting in their clonal expansion (7). Individuals with CH have a heightened risk for hematological malignancies, cardiovascular disease, and increased mortality from nonhematological cancers (7, 54, 55). However, the molecular mechanisms underlying the development of CH from mutated HSPCs remain elusive. Here, we showed that *Foxm1* haploinsufficiency promotes the expansion of HSPCs but eventually results in HSC exhaustion under different environmental stresses, recapitulating the development of CH and progression to MDS in patients. FOXM1 is frequently downregulated in patients with del(5q) MDS (32), and our data suggest a pathogenetic role of FOXM1 downregulation in patients with del(5q) MDS.

We previously showed that homozygous deletion of *Foxm1* results in a significant decrease of HSCs with a disrupted self-renewal capacity in vivo (32). However, we observed that *Foxm1* heterozygous mice exhibited an expansion of the HSC pool with a higher percentage of cycling cells and less quiescent stem cells, raising the possibility that Foxm1-downregulated HSCs have a dominant potential in hematopoiesis. As determined by in vivo competitive reconstitution assays, *Foxm1* heterozygous HSPCs gained a selective repopulation advantage over the control WT HSPCs at the first or first and second rounds of transplantation. However, this growth advantage exacerbates the functional HSC attrition, which leads to HSCs losing repopulation potential and exhaust over serial transplantation. In line with the results from the competitive repopulation assay, *Foxm1* heterozygous mice were more sensitive to 5-FU–induced depletion of hematopoietic cells, with a shorter survival time than WT control mice. This scenario of “expansion to exhaustion” in competitive assays resembles the evolution of CH to MDS. It was recently shown that some factors induce chronic stress, which contribute to the malignant transformation of hematopoietic clones (56, 57, 58). Chronic inflammation induced by low doses of LPS promoted the development of hematopoietic dysplasia in *Foxm1* heterozygous recipient mice but not control WT mice, characterized by multiple lineage cytopenia and the presence of dysplastic red cells in BM. Taken together, our results suggest that Foxm1 downregulation-mediated dysregulation of HSPC function may contribute to the initiation and development of del(5q) MDS in patients.

As determined by expression profiling analysis, multiple molecular pathways likely mediate the effects of *Foxm1* haploinsufficiency on HSCs. Particularly, downregulation of NR4a2 and upregulation of the lysosomal signaling pathway may contribute to *Foxm1* haploinsufficiency–induced HSC proliferation and expansion. As compared with the differentially expressed genes in *Foxm1*-KO HSCs (32), we found that a few genes including *NR4a2* were commonly downregulated in both *Foxm1* homozygous and heterozygous deletion HSCs as compared with the control HSCs (Supplemental Figure 4D). This suggests that *Foxm1* gene dosage is critical for regulation of gene expression of its downstream targets.

Consistent with the observation that *Foxm1* heterozygous deletion results in delays in DNA damage repair in HSPCs in response to chemical or irradiation-induced DNA damage, we showed that Parp1, which is involved in DNA damage repair (46, 47), is significantly downregulated in *Foxm1* heterozygous HSPCs. We demonstrated that Foxm1 regulates Parp1 expression by directly binding to its promoter region and that Parp1 at least partially mediates Foxm1 function in DNA damage repair in HSPCs. Interestingly, the expressions of *PARP1* and *FOXM1* are correlated, and both are downregulated in patients with del(5q) MDS, according to a public database (48). Moreover, long-term treatment with PARPi was found to induce secondary MDS/AML in therapy for ovarian cancer (27). Taken together, our results suggest that the Foxm1/Parp1-mediated DNA damage repair pathway may play an important role in the maintenance of genome stability in HSCs.

Foxm1 is a well-known oncogene that drives tumor cell proliferation in solid tumors (30). However, our recent research has revealed that Foxm1 is also crucial for maintaining the quiescence of leukemia stem cells (LSCs) in the MLL-AF9 mouse model. Loss of Foxm1 impairs LSC function, highlighting its critical role in MLL-rearranged leukemia (59). In addition, studies indicates that FOXM1 has an oncogenic role in AML (60) and can contribute to the development of drug resistance in cancers (61, 62). However, our study suggests that *Foxm1* downregulation may contribute to the pathogenesis of del(5q) MDS and AE9a-induced MPN in mice. While LSCs in AML possess enhanced self-renewal capacity, *Foxm1* overexpression may contribute to MLL-AF9–induced leukemogenesis by enhancing HSC self-renewal and quiescence. On the other hand, *Foxm1* downregulation may contribute to the pathogenesis of del(5q) MDS and AML1-ETO–induced MPN/AML by promoting the CH and influencing genome integrity at the early stage of diseases. Our findings demonstrate that precise regulation of Foxm1 expression is crucial for normal HSC function and both upregulation and downregulation of Foxm1 can contribute to the pathogenesis of myeloid malignancies through distinct cellular and molecular mechanisms.

In conclusion, our findings suggest that FOXM1 downregulation predisposes to hematopoietic dysplasia by deregulating the quiescence and self-renewal as well as genome stability of HSCs. Consequently, FOXM1 downregulation promotes the development of CH and the malignant transformation of HSCs in hematopoietic malignancies.

## Methods

### Mice.

The generation of mice with a targeted disruption of Foxm1 has been previously described (32). Briefly, *Foxm1*-floxed Tie2-Cre or Mx1-Cre mice were crossed with C57BL/6 (B6) mice. Mx1-Cre expression was induced by poly(I:C) HMW (InvivoGen), given by 3 i.p. injections at a dose of 10 mg/kg every other day. CD45.1 B6 mice were used as receipts in competitive assay and were purchased from The Jackson laboratory.

### Antibodies.

Information about the antibodies used in this study is provided in Supplemental Table 1.

### Cell sorting and flow cytometry.

Cells isolated from BM, spleen, thymus, and PB were lysed with ammonium-chloride-potassium (ACK) buffer. Single-cell suspensions were incubated with panels of fluorochrome-conjugated antibodies (Supplemental Table 1). For analysis of HSCs, whole BM cells were incubated with a lineage cocktail, including Gr1, B220, Ter119, CD19, Rat IgM, II-7R, and CD3, for 20 minutes, followed by staining with streptavidin-PE-Cy5 and antibodies against Sca-1 (PE), c-Kit (APC-Cy7), CD48(PE-Cy7), CD150 (APC). For G_0_ analysis with Ki67 and DAPI, 5 × 10^6^ BM cells were stained with surface markers as described before (32). After washing, the cells were fixed and permeabilized. Then, the cells were further stained with Ki67-FITC antibody and DAPI. For G_0_ analysis with Hoechst 33342 (catalog 62249) and Pyronin Y (catalog J61068.03) (Thermo Fisher Scientific), BM cells were incubated with 5 μg/mL Hoechst 33342 at 37°C. 45 minutes later, 1 μg/mL Pyronin Y was added to incubate for another 45 minutes. After that, the cells were stained with the HSC staining procedure described above. For the BrdU incorporation assay, mice were injected with 1 mg BrdU in 200 μL of PBS i.p. After 24 hours, BM cells were harvested and subjected to the HSC staining procedure described above. After that, the cells were stained with BrdU antibody and DAPI using the BD BrdU kit (catalog 559619).

For the analysis of γH2AX, cells were stained with HSC surface markers, followed by fixation and permeabilization as described above. After that, the cells were incubated with an antibody of γH2AX conjugated with Alexa Fluor 488 (CST, catalog 9719S) for 1 hour, followed by staining with DAPI for 1 hour. All data were analyzed by FlowJo version 9.3.3 analysis software (TreeStar). For cell sorting, after staining with antibodies against the cell surface markers, BM cells were sorted by MoFlo Astros cell sorter (Beckman Coulter).

### Colony formation assay.

Total BM cells from Tie2-Cre *Foxm1*^fl/+^(*n* = 2) and *Foxm1* ^fl/+^ mice (*n* = 2) were harvested and plated at 2 × 10^4^/mL in triplicate into Mouse Methylcellulose Base Media (R&D Systems) with 50 ng/mL mouse SCF and 10 ng/mL mouse IL-3 and IL-6 (5 × 10^4^ cell input for the first round, 15 × 10^4^ cell input for the second round and third round, triplicate for each group). Colonies were scored at 7–10 days after plating. Serial replating was performed after scoring.

### Virus production and infection.

For *FOXM1* shRNA lentivirus production, the shRNA constructs and the inducible knockdown system have been described in our previous work (59). Briefly, PLKO.1 Tet-On empty vector/*FOXM1*-Tet-On shRNAs combined with package plasmids (pMDG.2 and Δ8.91) were transfected into 293T cells by PEI. The medium containing lentivirus was harvested every 12 hours 4 times, starting 24 hours after transfection. Then, MDSL cells were mixed with the medium containing 8 μg/mL polybrene following by spinfection at 689 g for 3 hours. Spinfection was performed every 24 hours for twice.

For rescue assays, the pMSCV puro retroviral construct expressing PARP1 was generated by subcloning *PARP1* from pCMV-PARP1-3xFlag-WT (addgene 111575) with XhoI and HpaI. The overexpression construct or empty vector combined with PECO packaging plasmid was transfected into 293T cells. The medium containing retroviral particles was collected. Lin^–^c-Kit^+^ cells isolated from WT or Tie2-Cre *Foxm1^fl/+^* (Foxm1 HET) mice were mixed with PARP1-overexpressing retrovirus or empty vector virus. Spinfection was performed as described above.

For the AE9a-mediated AML model, empty vector or MSCV-Puro-GFP AML1-ETO9a plasmid (63) (donated by Dong-er Zhang, UCSD, La Jolla, California, USA), along with PECO, was mixed with PEI in medium and transfected into 293T cells. The supernatant medium was collected as described above. BM cells from WT or Foxm1 heterozygous KO mice with 5-FU injection were harvested and mixed with AE9a retrovirus and empty vector retrovirus separately. Then, spinfection was performed as above.

### 5-FU treatment.

Age- and sex-matched *Foxm1* heterozygous mice and control mice were challenged with 5-FU at a dose of 150 mg per kg body weight, once per week for 3 weeks, and the survival of mice was monitored daily. For analysis of γH2AX level, the mice were administrated 150 mg/kg 5-FU. 24 hours after injection, BM cells were isolated and analyzed with flow cytometry.

### BM transplantation.

For competitive repopulation assay, 1 × 10^6^ BM donor cells from Foxm1 heterozygous mice or control mice (CD45.2^+^) were mixed with 1 × 10^6^ CD45.1^+^CD45.2^+^ competitor cells in 100 μL PBS. These cells were transplanted into lethally irradiated CD45.1^+^ recipients. We harvested PB every month and checked the ratio by flow cytometry. To determine the long-term repopulating potential, every 4 months as 1 round, we sacrificed the mice and did serial transplantation.

For AE9a model, 8-week-old Foxm1 HET and *Foxm1^fl/+^*(WT) mice were injected with 150 mg/kg 5-FU (FRESENIUS KABI) i.p. After 5 days, BM cells were harvested and infected with retrovirus to transduce AE9a. After 2 rounds of infection, the infected cells were transplanted into sublethally irradiated B6 mice (6 Gy) by retro-orbital injection (1 × 10^6^ cells/mouse).

### LPS injection.

For primary mice, age- and sex-matched Tie2-Cre *Foxm1*^fl/+^ and *Foxm1*^fl/+^ mice were challenged with 10 mg/kg LPS (Sigma-Aldrich, L8643). BM cells were harvested from the mice injected with LPS after 24 hours. For transplantation mice, 2 × 10^6^ BM cells from *Foxm1* heterozygous KO mice or control mice were transplanted into lethally irradiated recipients. Engraftment efficiency was confirmed by checking PB 4 weeks after transplantation. These mice were subjected to i.p. injection of 1 mg/kg LPS twice per week for 2 months.

### Histology.

For histological analyses, murine organs were fixed in 3.7% formaldehyde immediately after necropsy. H&E staining of fixed tissues was performed by the Robert H. Lurie Comprehensive Cancer Center of Northwestern University.

### RNA-Seq.

BM cells were harvested from 3 pairs of *Foxm1* heterozygous mice and control mice. After staining with HSC surface markers, LT-HSC cells (CD150^+^CD48^–^LSK) were sorted as described above. Total RNA from LT-HSC cells was isolated by Trizol (Invitrogen) at a yield of around 5–10 ng. The library was prepared with the Library Construction Kit (Clontech). RNA-Seq was performed on a Illumina HiSeq 3000 system with 50 bp single-read mode by Clinical Microarray Core at UCLA (Los Angeles, California, USA). The sequencing depth was 50 million reads per sample. Quality check for low-quality reads and adapters in raw reads data were reported and trimmed using FastQC tool. The filtered reads were aligned to GRCm38 (mm10) using STAR V2.7.2b (64). Gene set enrichment analysis was performed with GSEAv2.0 software, which is available from the Broad Institute (http://www.broad.mit.edu/gsea).

### RT-PCR and CUT&RUN assay.

Sorted HSCs from WT or *Foxm1* HET mice were lysed in TRIzol. RNA was extracted by phenol-chloroform. Total RNA was then amplified with the Ovation Pico WTA System V2. For the CUT&RUN assay, EpiCypher CUTANA CUT&RUN Protocol v1.7 has been followed. Rabbit IgG^–^ control antibody (EpiCypher, 13-0042), H3K4me3 antibody (EpiCypher, 13-0041), Mouse IgG negative control antibody (Cell Signaling Technology, 37988S), and Foxm1 antibody (Santa Cruz, sc-376471) were used. Briefly, 500,000 Lin^–^c-Kit^+^ cells isolated from WT mice were washed and mixed with concanavalin A beads (EpiCypher, 21-1401) for 10 minutes at room temperature. Then, cell/bead conjugates were resuspended with antibody buffer, including 0.5 μg of the indicated antibody and 0.003% digitonin. After overnight incubation at 4°C, the cell/bead conjugates were washed and incubated with CUTANA pA/G-MNase (Epicypher,15-1016) for 2 hours at 4°C. To stop the digestion of pA/G-MNase, the stop buffer was added followed by the purification of released chromatin fragments using the CUTANA DNA Purification Kit (Epicypher, 14-0050). RT-PCR was performed on an Applied Biosystems 7500 thermocycler using the following primer sequences listed in Supplemental Table 2 and analyzed via the ΔΔCT method.

The MDSL cell line, which was originally established by Kaoru Tohyama’s laboratory (65) (Kawasaki Medical School, Okayama, Japan), was provided by Daniel Starczynowski (University of Cincinnati College of Medicine, Cincinnati, Ohio, USA). To evaluate the level of PARP1 in MDSL with FOXM1 knockdown, MDSL cells were transduced with *FOXM1* shRNA by spinoculating with pLKO.1 Tet-On virus as we showed previously (59). After being selected with 1 μg/mL puromycin for 4 days, the cells were treated with 2 μg/mL doxycycline for 24 hours to induce *FOXM1* shRNA expression. All results are from 2 or 3 independent experiments.

### Immunofluorescence microscopy.

Whole BM cells were harvested from mice as indicated and incubated with the Biotin Mouse Lineage Depletion Cocktail (in house; see Supplemental Table 1), including antibodies Gr1, B220, Ter119, CD19, Rat IgM, II-7R, and CD3 for 20 minutes. After washing away the excess antibodies, the cells were suspended with isolation buffer and incubated with magnetic beads (Invitrogen, catalog 11047) for 30 minutes at 4°C with gentle rotation. After depletion of lineage cells, the BM cells were enriched with CD117 MicroBeads (catalog 130-091-224). For immunostaining, the isolated Lin^–^c-Kit^+^ BM cells were resuspended in fresh medium and dropped on a coverslip precoated with ploy-L-Lysine. After 1 hour, 4% PFA was added to fix the cells and 1% Triton X-100 was used to permeabilize the membranes followed by 1-hour incubation of 5% BSA at room temperature. γH2AX antibody (Invitrogen, MA1-2022) in 1% BSA was added and incubated at 4°C overnight. After washing with cold 0.2% Triton X-100, the coverslips were incubated with secondary antibody conjugated with Alexa Fluor 488 (Jackson immune Research, 115-545-003) for 1 hour at room temperature followed by staining DAPI for 5 minutes. Images were acquired in a Leica-TCS microscope. To estimate the number of γH2AX foci^+^ cells, 200–500 cells were scored in each condition.

### DNA damage assays.

Lin^–^ BM cells were isolated from mice and plated on the precoated coverslip. One hour later, the cells were subjected to 0.5 Gy irradiation. At different time points, immunostaining was performed using the γH2AX antibody as described above. To check the kinetics of γH2AX in a different population, total BM cells were exposed to 0.5 Gy irradiation and collected at different time points as indicated. FACS was performed to analyze the γH2AX level as described above.

### Comet assay.

The Lin^–^c-Kit^+^ BM cells were isolated as described above. Comet assay was performed using the CometAssay Kit(4250-050-K) according to the manufacturer’s instructions. Images were acquired by Leica DM2500 and analyzed by CometScore 2.0 (http://rexhoover.com/index.php?id=cometscore).

### Statistics.

Results are presented as mean ± SD. Statistical analysis was performed with 2-tailed Student’s *t* test or 2-way ANOVA with Tukey’s multiple-comparison test using GraphPad Prism v8.0 software. Survival curves were compiled using the Kaplan-Meier algorithms of GraphPad Prism, and significance was assessed using the log-rank (Mantel-Cox) test. Correlation was calculated according to Spearman’s statistical analysis by GraphPad Prism. The colony-forming assay, qRT-PCR, immunostaining, and cell culture experiments were done with 2–3 technical replicates and repeated at least 2–3 times. *P* values equal to or less than 0.05 were considered statistically significant.

### Study approval.

All experiments and procedures were approved by the University of Florida Institutional Animal Care and Use Committee.

### Data availability.

RNA-Seq raw data in.fastq format has been uploaded into the NCBI Sequence Read Archive (SRA) database (accession SRP181055). The raw and normalized gene expression data have been deposited in NCBI GEO database (accession GSE208564). Values for all data points in graphs are reported in the supporting data values file.

## Author contributions

ZQ and CY designed the experiments. CY, YS, and FY performed experiments and interpreted the results. HN provided histological analysis. YH and CY analyzed RNA-Seq data. ZQ, CY, and AQ contributed to preparation of the manuscript. FY provided advice and new reagents/analytic tools. All authors provided critical review of the manuscript.

## Supplementary Material

Supplemental data

Supplemental table 1

Supplemental table 2

Supporting data values

## Version 1 08/01/2023

Electronic publication
