# Supplementary material for: Foxm1 haploinsufficiency drives clonal hematopoiesis and promotes a stress-related transition to hematologic malignancy in mice
Source: J Clin Invest. 2023 Aug 1;133(15):e163911. doi: 10.1172/JCI163911 (PMC10378147; doi:10.1172/JCI163911)
Supplement: Supplemental data [file jci-133-163911-s077.pdf]

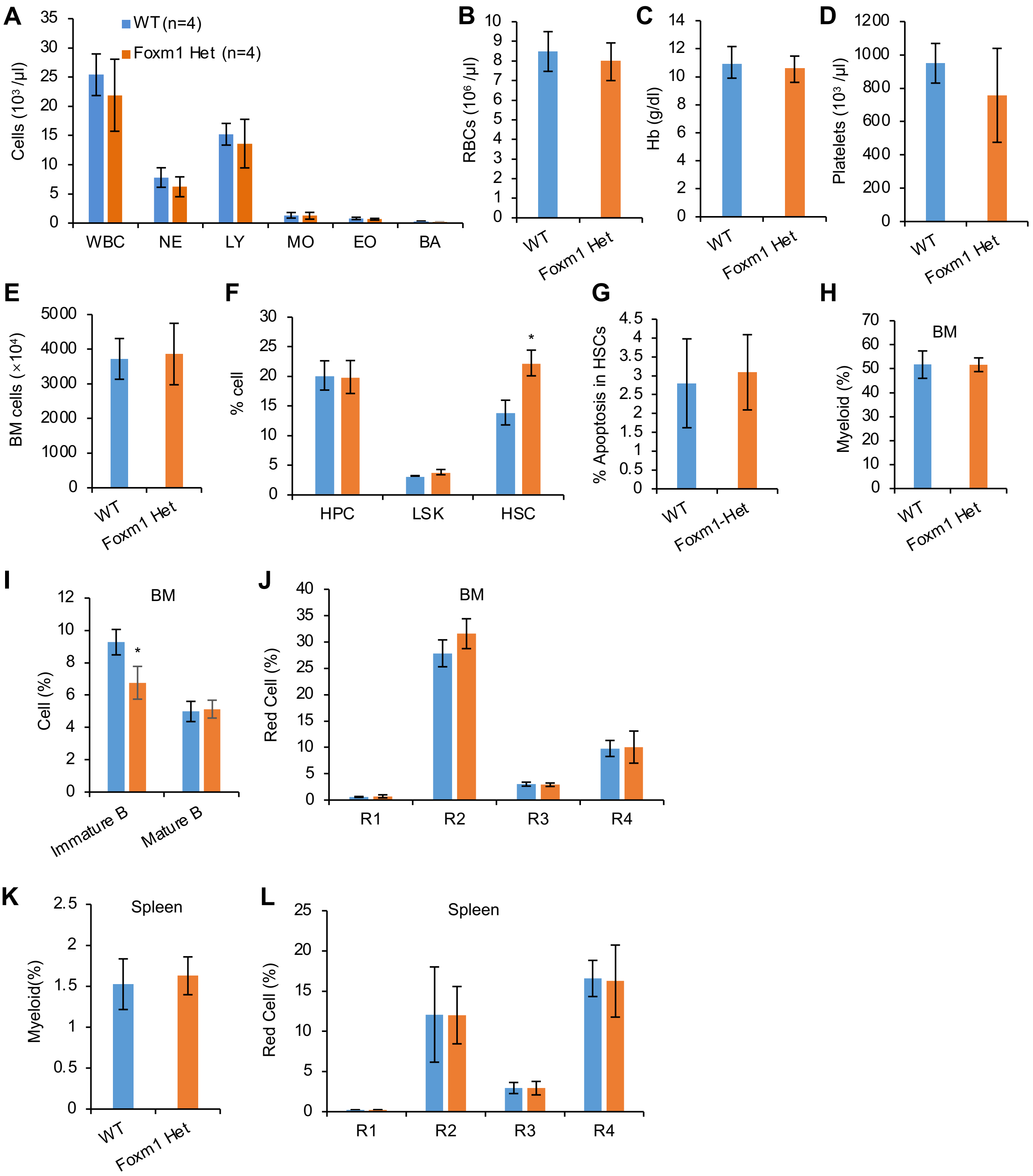

Supplemental Figure 1. Characteristic of *Foxm1* heterozygous KO mice. (A) Absolute number of white blood cells (WBC), neutrophils (NE), lymphocytes (LY), monocytes (MO), eosinophils (EO) and basophils (BA), (B) red blood cells (RBC), (C) concentration of hemoglobin (Hb) and (D) platelets (PLT) in peripheral blood from *Foxm1*-heterozygous KO mice(n=4) and WT control mice(n=4). (E)Total BM cells from *Foxm1* heterozygous KO mice (n=5) and WT control mice(n=5). (F) Frequency of HPC, LSK and HSC in BM from *Foxm1* heterozygous KO mice(n=3) and WT control mice(n=3). (G) Flow cytometry analysis shows the percentage of apoptosis in HSCs. WT, n=4; *Foxm1* Het, n=5. The frequency of mature cells in BM including Myeloid (H), B cell(I) and Red cell(J). The frequency of mature cells in spleen including Myeloid(K) and Red cell(L). n=4 for each group. Data are presented as mean ± SD, 2-tailed Student's t test; *$p < 0.05$, **$p < 0.01$, ***$p < 0.001$.

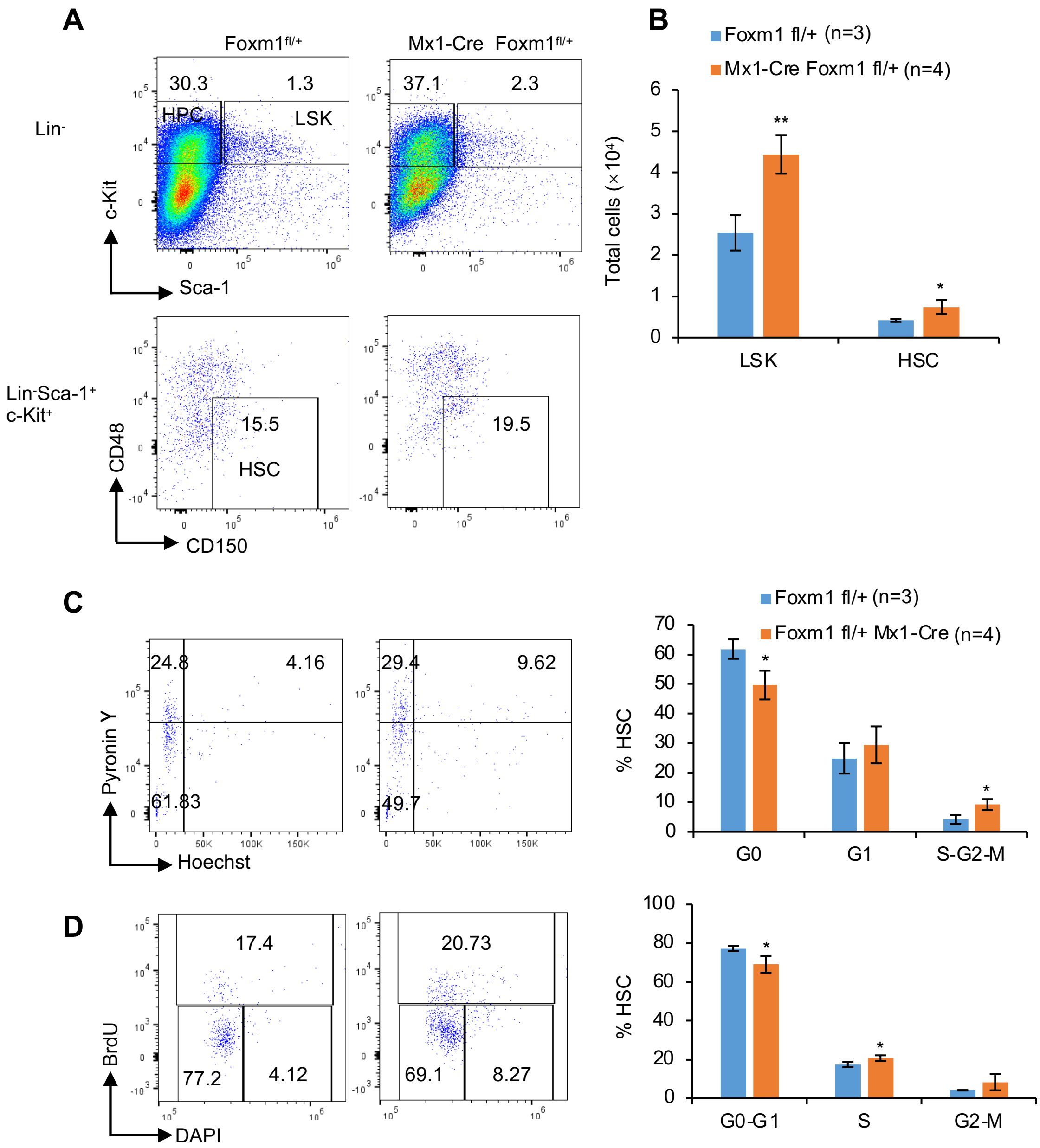

Supplemental Figure 2. ***Foxm1*** **knockout by Mx1-Cre Increases active HSCs and expands the HSC pool in mice.** (A) Flow cytometric analysis of LSK and HSC in BM cells from *Foxm1*$^{fl/+}$ mice(n=3) Mx1-Cre *Foxm1*$^{fl/+}$ mice (n=4) after 1 month of pIpC injection. (B) A total number of LSK cells and HSCs in BM. (C-D) FACS analysis of cell cycle in HSCs by different staining methods as indicated. Data are presented as mean ± SD, 2-tailed Student's t test; *$p < 0.05$, **$p < 0.01$, ***$p < 0.001$.

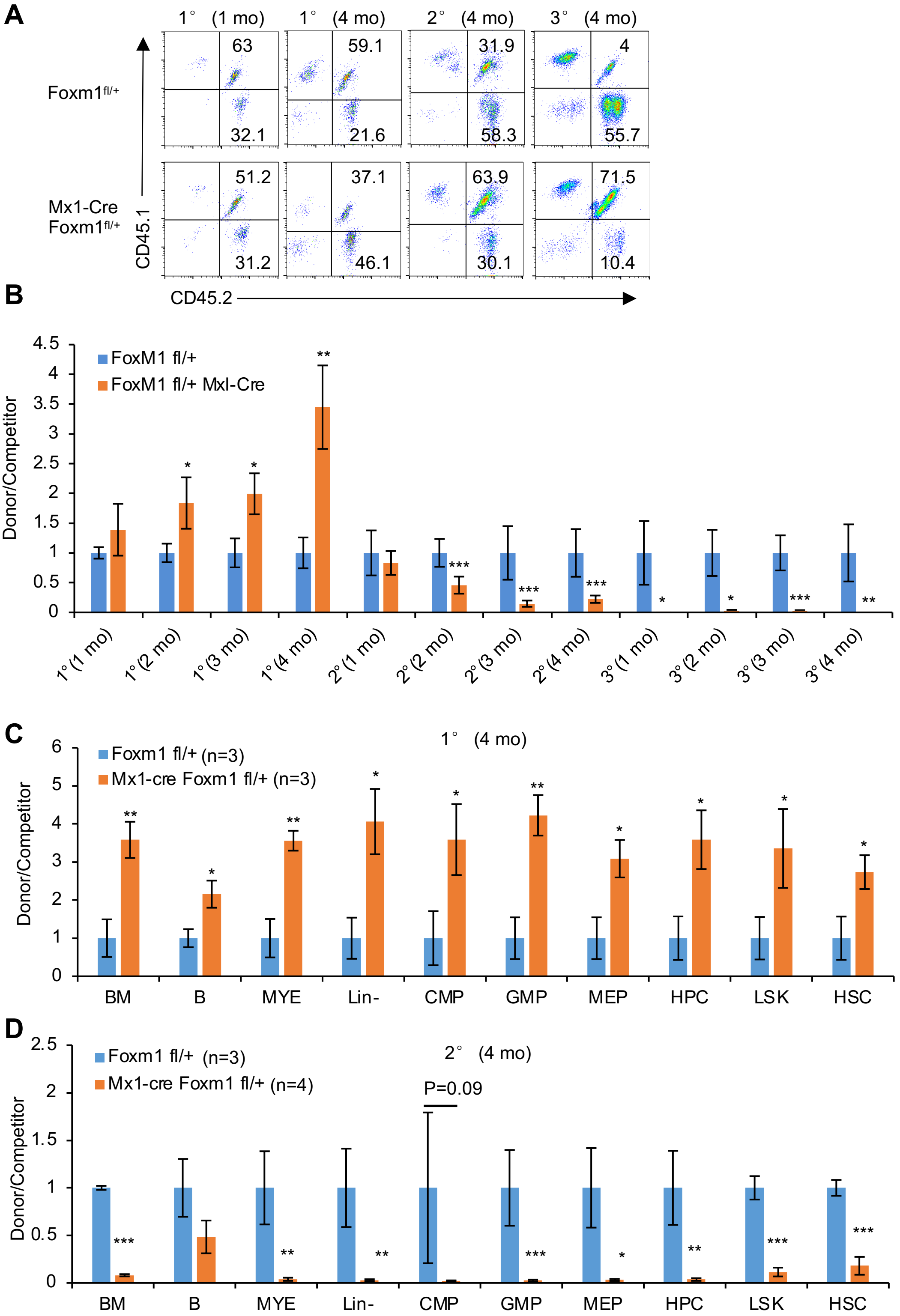

Supplemental Figure 3. Mice transplanted with BM cells from Mx1-Cre *Foxm1*$^{fl/+}$ mice show a transient hypercellular marrow and eventual HSC exhaustion. (A) Flow cytometric analysis shows frequency of CD45.2$^+$ CD45.1$^-$(donor cells) and CD45.1$^+$CD45.2$^+$ (competitor cells) in peripheral blood from chimeric mice reconstituted with BM cells from *Foxm1*$^{fl/+}$ or Mx1-Cre *Foxm1*$^{fl/+}$ mice at 1st month after 1st transplantation or 4th month after each round of transplantation. (B) Ratio of Donor-derived cells(CD45.2$^+$ CD45.1$^-$) to Competitor-derived cells(CD45.2$^+$ CD45.1$^+$) in peripheral blood. For WT group, n=3, 6-7, 5 for 1°, 2° and 3° transplantation respectively; for *Foxm1* HET group, n=4-5, 9-10, 3-5 for 1°, 2° and 3° transplantation respectively. (C) Ratio of Donor-derived cells to Competitor-derived cells in BM at 4th month after 1st round transplantation. (D) Ratio of Donor-derived cells to Competitor-derived cells in BM at 4th month after 2nd round transplantation. Data are presented as mean ± SD, 2-tailed Student's t test; *$p < 0.05$, **$p < 0.01$, ***$p < 0.001$.

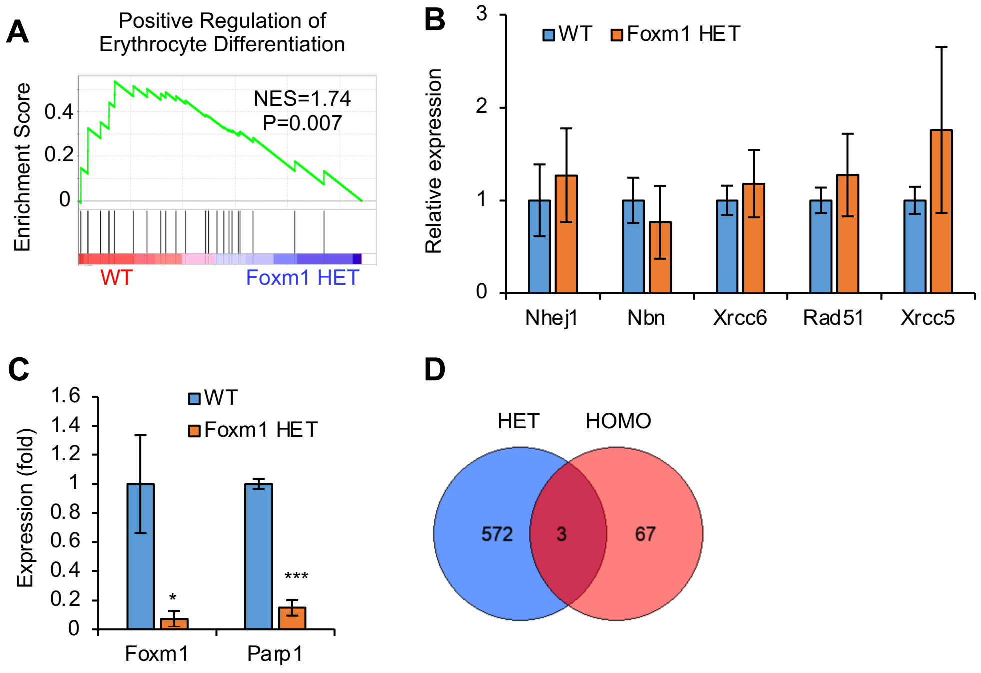

Supplemental Figure 4. Molecular signaling pathways related with Foxm1 in hematopoiesis. (A) GSEA polts showing negative association with pathway in "Positive Regulation of Erythrocyte Differentiation" in Foxm1 HET HSCs. (B) RT-PCR analysis shows the expression level of genes in DNA damage repair pathway in HSCs. n=2-3 for each group. (C) RT-PCR shows Parp1 downregulated in 5-FU-enriched progenitor populations from Tie2-Cre; *Foxm1*$^{fl/+}$ and control mice injected with 5-FU for 5 days. n=3 for each group. (D) Venn diagram of differentially expressed genes (P value <0.05, FC >2) in *Foxm1* heterozygous HSC(RNA Seq) versus *Foxm1* homozygous HSCs(Microarray). Data are presented as mean $\pm$ SD, 2-tailed Student's t test; *p < 0.05, **p < 0.01, ***p < 0.001.

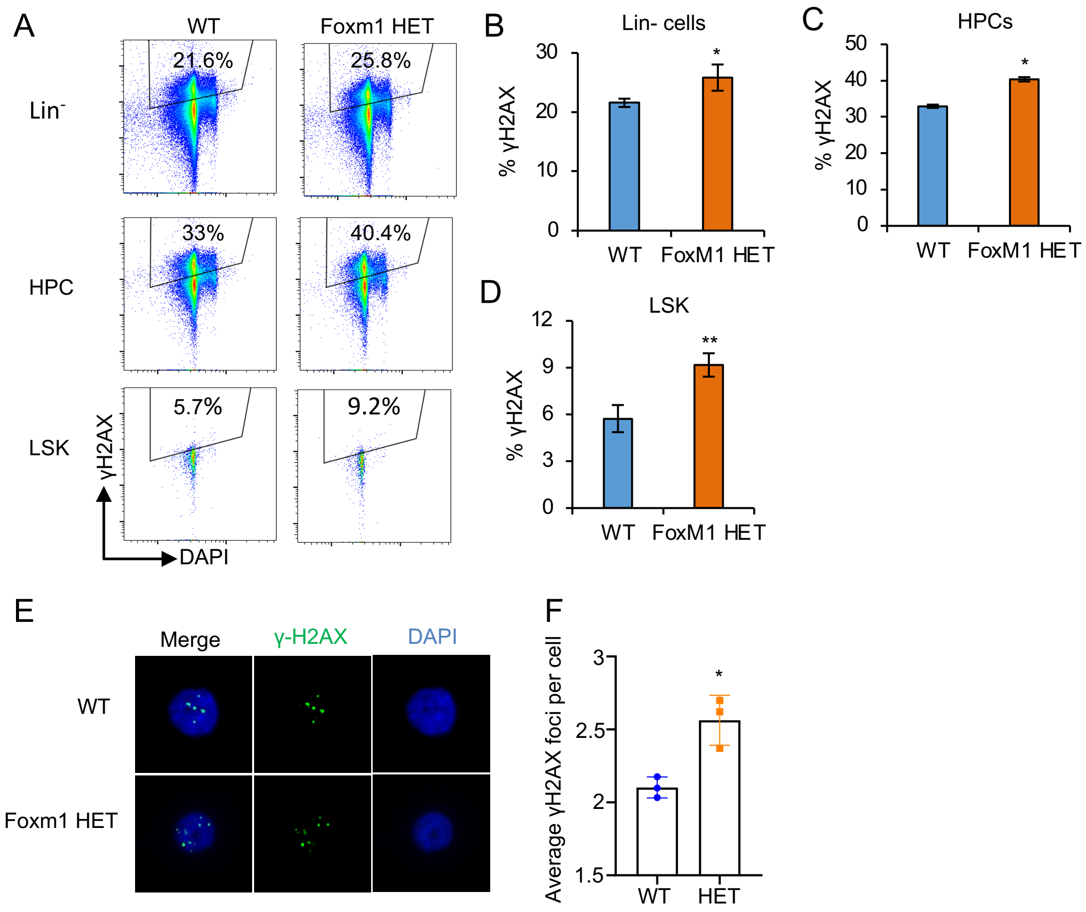

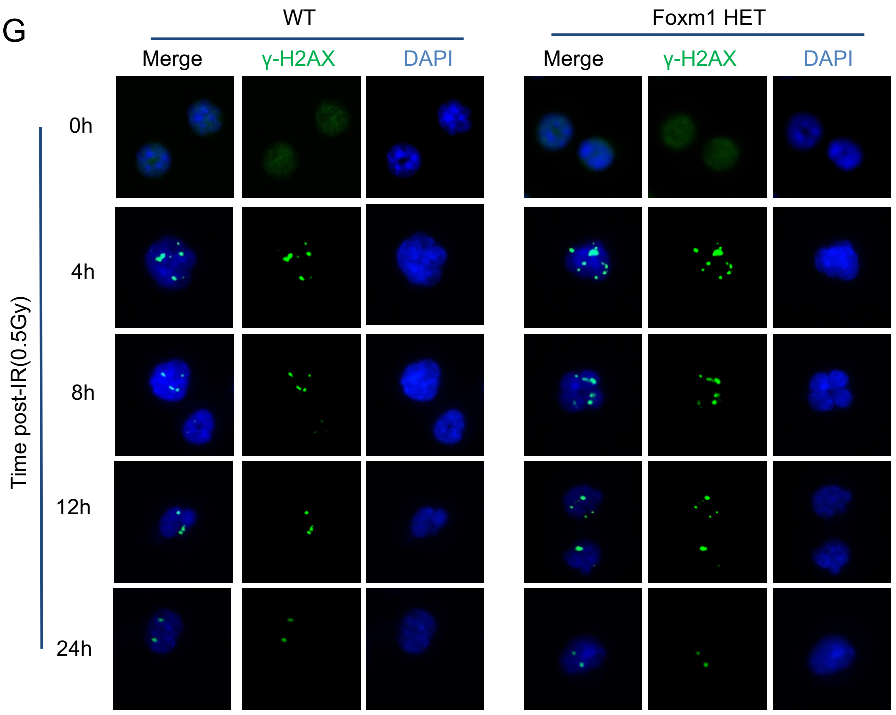

Supplemental Figure 5. *Foxm1* haploinsufficiency impaired DNA damage repair in hematopoietic progenitor/stem cells upon 5-FU challenge and irradiation.
(A)Representative flow cytometric plots show the percentage of γH2AX–positive cells in Lin$^-$ cells, HPCs (Lin$^-$c-Kit$^+$Sca-1$^-$) and LSKs from Tie2-Cre *Foxm1*$^{fl/+}$ and control mice 24 hours after administering 150mg/kg 5-FU. (B-D)Histogram show the percentage of γH2AX–positive cells in different populations as indicated. WT, n=3; Foxm1 Het, n=3. (E)Representative images of γH2AX foci in Lin$^-$ BM cells isolated from Tie2-Cre *Foxm1*$^{fl/+}$ and control mice after 24 hours post-5-FU-injection.(F)Quantification of the average number of γH2AX foci/cell in Lin$^-$ BM cells from mice injected with 5-FU after 24 hours. WT, n=3; Foxm1 Het, n=3. (G)Representative images of γH2AX foci in Lin$^-$ BM cells isolated from Tie2-Cre *Foxm1*$^{fl/+}$ and control mice at different time point post irradiation(0.5 Gy). n=3 for each group. Data are presented as mean ± SD, 2-tailed Student's t test; *$p < 0.05$, **$p < 0.01$, ***$p < 0.001$.

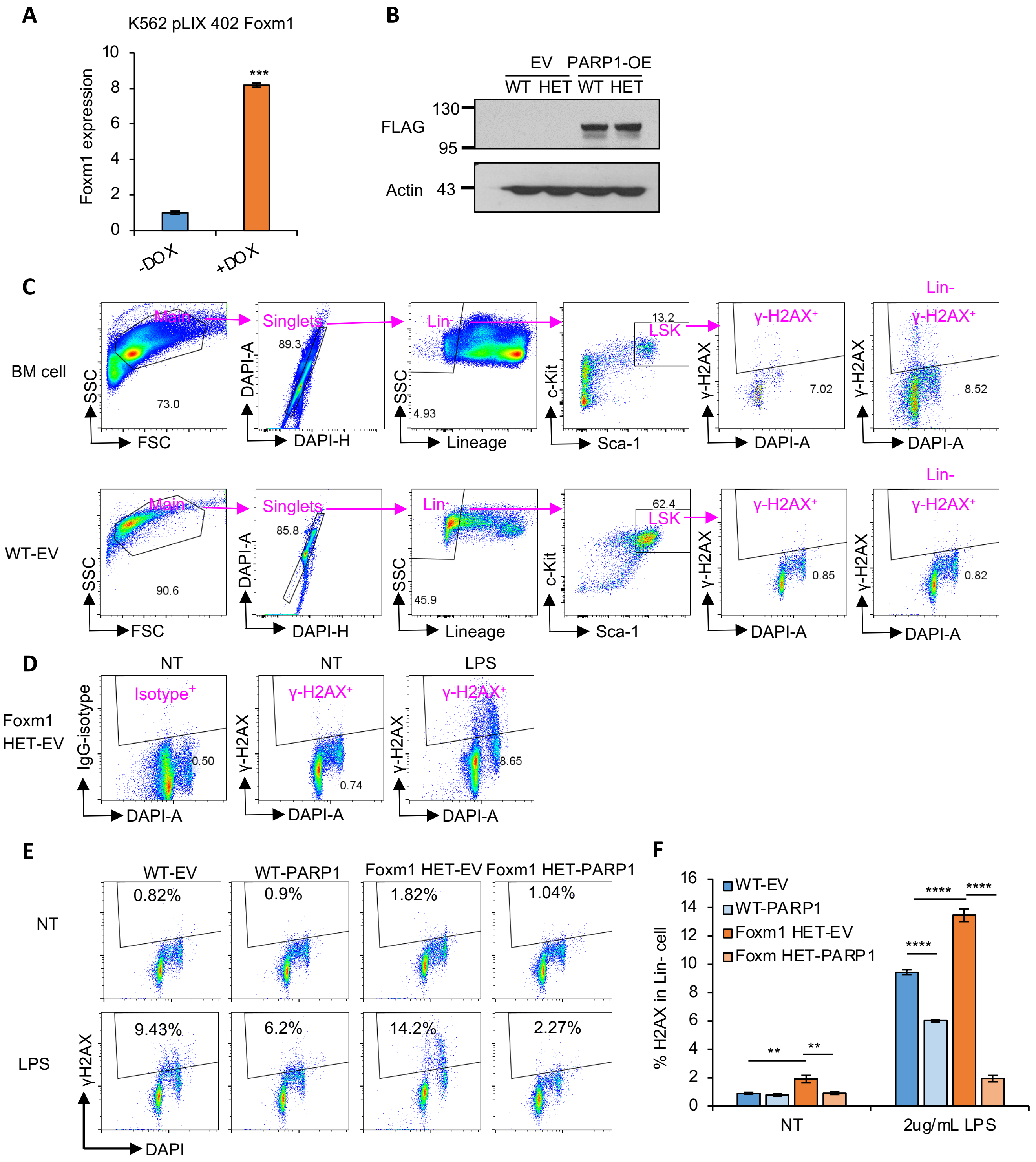

Supplemental Figure 6. PARP1 rescues the defect on DNA damage repair induced by *Foxm1* haploinsufficiency. (A) In the luciferase assay, *Foxm1* was overexpressed by the induction of Doxycycline in K562 pLIX 402 Foxm1 cells. (B) Western blotting shows that PARP1-Flag was overexpressed in Lin$^-$c-Kit$^+$ BM cells transduced PARP1. C) Flow plots show the gating strategy for analyzing γ-H2AX level in Lin$^-$c-Kit$^+$ BM cells after LPS treatment. (D) Isotype IgG was used as negative control to set up the gating strategy for analyzing the level of γ-H2AX. (E) Representative FACS analysis of γH2AX in Lin$^-$ population for the cells treated with or without LPS. (F) Quantification of the frequency of γH2AX in Lin$^-$ population from the cells as indicated after LPS treatment. n=3 for each group. Data are presented as mean ± SD, 2-tailed Student's t test or 2-way ANOVA with Tukey's multiple-comparison test. *p < 0.05, **p < 0.01, ***p < 0.001, ****p < 0.0001.

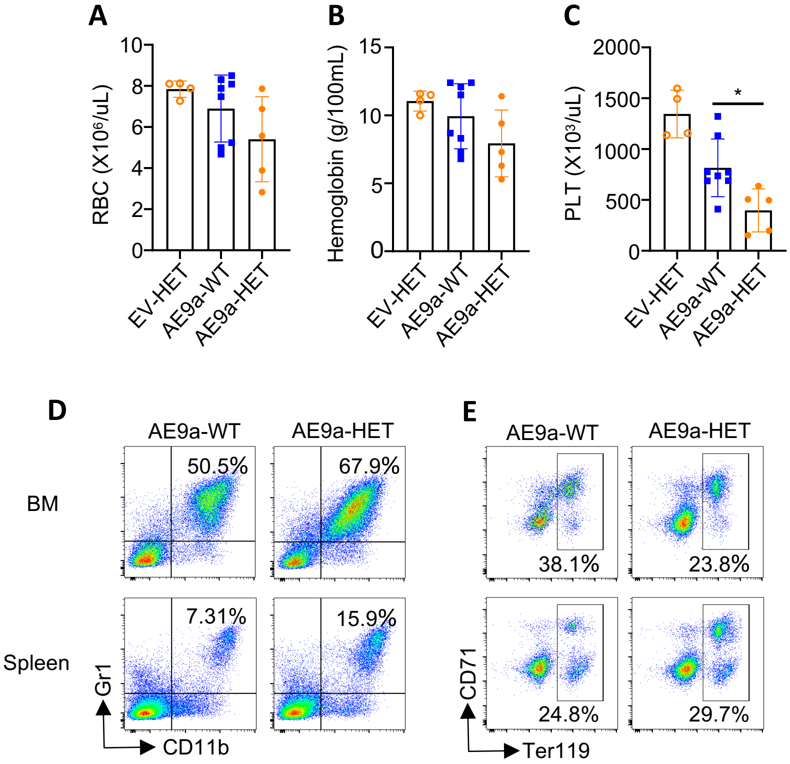

Supplemental Figure 7. *Foxm1* haploinsufficiency promotes AML1-ETO9a (AE9a)-induced AML progression in vivo. (A)-(C) Absolute number of red blood cells (RBC), concentration of hemoglobin (Hb) and platelets (PLT) in peripheral blood from receipt mice transplanted with Foxm1 heterozygous BM cells transduced empty vector(EV-HET), WT BM cells transduced with MSCV-PIG-AE9a(AE9a-WT) and *Foxm1* heterozygous BM cells transduced with MSCV-PIG-AE9a(AE9a-HET). n=4 for EV-HET group, n=8 for AE9a-WT group, n=5 for AE9a-HET group. Data are presented as mean ± SD, 2-tailed Student's t test. *p < 0.05, **p < 0.01, ***p < 0.001. (D)-(E) Representative flow plots show the frequency of Myeloid cells (D) and Red blood cells (E) in GFP⁺ BM cells and splenic cells from mice transplanted with AE9a-WT and AE9a-Foxm1 HET cells.
